# Intranasal delivery of a COBRA-based protein vaccine adjuvanted with c-di-AMP enhances breadth of protective immune responses against seasonal influenza viruses

**DOI:** 10.3389/fimmu.2026.1876199

**Published:** 2026-08-21

**Authors:** Camila C. S. Caetano, David A. Prots, Matthew H. Thomas, Dylan R. Micallef, Robert A. Richardson, Ted M. Ross

**Affiliations:** 1 Department of Infection Biology, Global Center for Pathogen Research and Human Health, Cleveland Clinic, Cleveland, OH, United States; 2 Florida Research and Innovation Center, Cleveland Clinic, Port Saint Lucie, FL, United States; 3 Center for Vaccines and Immunology, Department of Infectious Diseases University of Georgia, Athens, GA, United States

**Keywords:** adjuvant, cellular immunity, hemagglutinin, humoral immunity, influenza, neuraminidase

## Abstract

Seasonal influenza vaccines provide variable protection against antigenically drifted influenza A (H1N1 and H3N2) and influenza B viruses. Broadly protective approaches using computationally optimized broadly reactive antigens (COBRAs) can enhance immunity against diverse strains. Bis-(3′,5′)-cyclic dimeric adenosine monophosphate (c-di-AMP) is a mucosal adjuvant that boosts vaccine-induced immune responses. Here, we hypothesized that intranasal multivalent COBRA hemagglutinin (HA) and neuraminidase (NA) vaccination with c-di-AMP would induce broad humoral and cellular immunity in mice. Immunologically naïve and pre-immune DBA/2J mice were intranasally immunized with pentavalent vaccine formulations containing COBRA recombinant HA and NA or corresponding wild-type antigens. Formulations were administered at low or high antigen doses, with or without the mucosal adjuvant c-di-AMP. Humoral and cellular immune responses were evaluated, along with protection following influenza A (H1N1 and H3N2) and influenza B viral challenge. Pentavalent COBRA HA/NA intranasal vaccination adjuvanted with c-di-AMP induced strong humoral and cellular immune responses in murine models. Vaccination induced robust, dose-dependent total IgG and hemagglutination inhibition (HAI) titers, along with high frequencies of antigen-specific antibody-secreting cells and interferon-γ (IFN-γ)-producing cells in pre-immune mice. Naïve mice were also seroprotected mainly against H1N1 at higher vaccine doses. Following lethal challenge with H1N1, H3N2, and influenza B viruses, vaccinated mice exhibited reduced weight loss, decreased lung viral titers, and enhanced survival, with the strongest protection observed in adjuvanted groups across both naïve and pre-immune models. These findings highlight the value of combining broadly protective COBRA vaccine candidates with advanced mucosal adjuvants to enhance efficacy against antigenically drifted influenza virus strains.

## 1 Introduction

Current seasonal influenza vaccines remain the primary strategy to reduce influenza burden, yet in adults, these vaccines remain suboptimal, typically ranging from 30% to 60% efficacy (1). Protection is largely confined to strain-specific antibodies targeting the variable hemagglutinin (HA) head domain. Because of the co-circulation of influenza A (H1N1 and H3N2) and influenza B viruses (IBVs), combined with their propensity for continuous antigenic drift, vaccine-strain mismatches occur frequently, necessitating periodic updates to vaccine compositions (2). These mismatches significantly reduce vaccine effectiveness, particularly when circulating viruses diverge from vaccine components (3).

To address these limitations, next-generation immunogens designed using the computationally optimized broadly reactive antigen (COBRA) approach incorporate conserved epitopes within HA and neuraminidase (NA), generated through multilayered consensus sequence alignment across multiple influenza subtypes (4, 5). While COBRA HA vaccines consistently elicit broader responses than wild-type (WT) comparators (5–10), the inclusion of NA in these formulations further promotes breadth and more durable protection (11–14). Compared to HA, the NA proteins harbor more conserved epitopes, elicit cross-reactive antibodies, and correlate with protection (15, 16), supporting NA as a complementary target that can strengthen breadth when included alongside HA.

Most licensed influenza vaccines are administered intramuscularly, which primarily induces systemic immune responses (17). Intranasal vaccines enhance protection by inducing both mucosal and systemic immunity, and represent a promising next-generation strategy for influenza virus control (18, 19). This vaccination route establishes tissue-resident memory (Trm) B and T cells within the respiratory tract, serving as a local immune priming site while still allowing antigen-specific lymphocytes to disseminate and contribute to systemic immunity (20). Thus, mucosal vaccines extend well beyond the mucosal site of administration.

Conversely, soluble protein antigens are typically poorly immunogenic when administered intranasally; therefore, adjuvants are often included to enhance immune responses and improve overall vaccine efficacy. In the present study, we evaluated the effectiveness of a pentavalent COBRA HA and NA vaccine formulation co-administered with or without the interferon gene (STING) agonist bis-(3′,5′)-cyclic dimeric adenosine monophosphate (c-di-AMP) adjuvant via the intranasal route in mice. Cyclic dinucleotides (CDNs), such as c-di-AMP, have emerged as effective adjuvants due to their ability to induce robust humoral and cellular memory immune responses across both systemic and mucosal compartments (21). By combining the pentavalent COBRA platform with c-di-AMP via the intranasal route, the vaccine induced robust, cross-reactive immunity capable of protecting against antigenically diverse influenza viruses.

Here, key factors that shape influenza vaccine-induced immunity were examined, including the vaccine design, antigen dose, route of administration, pre-existing anti-influenza immunity, and adjuvant use. Our findings indicate that pentavalent COBRA HA/NA proteins formulated with a mucosal adjuvant elicited robust antibody titers, resulting in broadly seroprotective humoral immune responses against influenza A and B viruses. The formulation also induced stronger antigen-specific cellular immune responses than the unadjuvanted COBRA vaccine with higher doses consistently producing the greatest protective responses. Crucially, the inclusion of c-di-AMP induced protective immune responses even at lower antigen doses that otherwise fail to elicit such responses when administered without an adjuvant. The immune responses correlated with protection and pre-existing immunity to historical influenza viruses are a critical determinant of vaccine efficacy. These parameters provide a framework for optimizing universal influenza vaccine designs.

## 2 Materials and methods

### 2.1 Vaccine antigens

COBRA HA and NA antigens representing the H1 (Y2), H3 (NG7), IBV (BC17), N1 (N1I), and N2 (N2B) subtypes were generated and used as recombinant vaccine antigens. The design and development of these COBRA antigens have been described previously (4, 7–9, 22, 23). Briefly, full-length WT HA or NA sequences for each subtype were collected and aligned using Geneious v11.1.5. Primary consensus sequences were generated from clusters of WT sequences with high sequence similarity, and these sequences were subsequently used to generate secondary consensus sequences. This iterative consensus-building process was repeated until a single final COBRA consensus sequence was derived. In addition to the COBRA antigens, recombinant WT influenza proteins were also generated for use in vaccination. These proteins represented the HA and NA from the H1N1 A/California/07/2009 (CA/09) and HA and NA from the H3N2 A/Hong Kong/4801/2014 (HK/14), and the HA from the IBV B/Phuket/3073/2013 (PH/13). The resulting HA and NA sequences were modified for recombinant protein expression by truncating the transmembrane domain to generate soluble recombinant proteins. The soluble proteins were collected from cell culture supernatants and purified using His-tag affinity via chromatography as previously described (24). The purified rHA/rNA proteins were used to coat enzyme-linked immunosorbent assay (ELISA) plates for serological assays and formulated for intranasal vaccination with or without adjuvant cyclic di-adenylate monophosphate (c-di-AMP) (InvivoGen, San Diego, CA).

### 2.2 Viruses

Influenza A(H1N1), A(H3N2), and IBVs were obtained from the Influenza Reagents Resource (IRR), BEI Resources, the United States Centers for Disease Control and Prevention (CDC), or Virapur (San Diego, CA, USA). Viruses were propagated either in embryonated chicken eggs or in Madin–Darby canine kidney (MDCK) cell cultures as previously described (25). After harvest, viruses were aliquoted into individual tubes and stored at −80°C for single-use applications. Hemagglutination activity of the A(H1N1) and IBVs was determined using 0.8% turkey red blood cells (TRBCs) (Lampire Biological Laboratories, Pipersville, PA, USA), and the HAI activity of the A/H3N2 viruses was determined using 0.8% guinea pig red blood cells (GPRBCs) (Lampire Biological Laboratories, Pipersville, PA, USA) supplemented with 20 nM oseltamivir (Aobious, Gloucester, MA, USA). The H3N2 influenza virus A/Panama/2007/1999 (PN/99), the H1N1 virus A/Singapore/6/1986 (SG/86), and the IBV virus B/Hong Kong/330/2001 (HK/01), were used to establish pre-immunity in mice as previously described (11, 12, 26). For challenge infections, mice were infected with the H1N1 influenza virus A/Brisbane/02/2018 (BR/18), the H3N2 virus A/Switzerland/9715293/2013 (SW/13), or the IBV B/Washington/02/2019 (WA/19).

For hemagglutination inhibition (HAI) assays, panels of H1N1, H3N2, and IBVs were used. The H1N1 virus panel included BR/18, A/Guangdong/SWL1536/2019 (GD/19), and A/Victoria/2570/2019 (VC/19). The H3N2 virus panel included SW/13, A/South Australia/34/2019 (SA/19), A/Hong Kong/2671/2019 (HK/19), and A/Tasmania/503/2020 (TS/20). The IBV panel (Victoria lineage) included B/Brisbane/60/2008 (BR/08), B/Colorado/06/2017 (CO/17), and WA/19.

### 2.3 Mouse vaccinations and infections

Female DBA/2J mice (6–8 weeks old; The Jackson Laboratory) were housed in microisolators with food and water available *ad libitum* and acclimated upon arrival. Mice were housed in microisolator units with *ad libitum* access to food and water throughout the study. All procedures were USDA-compliant and approved by the Cleveland Clinic IACUC (#00003027). Parallel naïve and pre-immune influenza cohorts were established. Pre-immune mice were intranasally infected on day −30 with SG/86 (H1N1), PN/99 (H3N2) at 5 × 10^5^ plaque-forming units (PFU) each, and HK/01 (IBV) at 1 × 10² PFU in 50 µL of PBS (phosphate-buffered saline) to establish immune history. Weight and clinical signs were monitored for 14 days, and seroconversion was confirmed. Naïve and pre-immune mice were randomized into nine vaccination groups. Vaccines contained either a pentavalent COBRA formulation [Y2 HA (H1), NG7 HA (H3), BC17 HA (IBV), N1I (N1), and N2B (N2)] or a WT formulation (CA/09 HA, HK/14 HA, PH/13 HA, CA/09 NA, and HK/14 NA) at 0.1 or 1 µg per antigen in PBS (Corning, Tewksbury, MA, USA). Adjuvanted groups received 5 µg of c-di-AMP (InvivoGen, San Diego, CA, USA). Vaccines were administered intranasally (50 µL) on days 0 and 28. Blood was collected from the submandibular vein on days 14 and 42. Serum was isolated from the blood by centrifugation at 5,000 rpm for 5 min using BD Microtainer blood collection tubes (Becton Dickinson, Franklin Lakes, NJ, USA) and stored at −20°C. Spleens from pre-immune mice that received 1 µg dose of each antigen were collected on day 35. On day 56, mice were intranasally challenged with BR/18 (3 × 10^4^ PFU), SW/13 (4.75 × 10^6^ PFU), or WA/19 (1 × 10^3^ PFU), and monitored for 12 days. Challenge doses for each viral strain were selected based on prior dose-titration experiments in DBA/2J mice to achieve lethal dose in unvaccinated mock control animals within the 12-day observation period, as described previously (11, 27). Differences in absolute PFU values among subtypes reflect differences in the intrinsic pathogenicity of each virus strain, rather than variations in challenge dose. Three mice per group were euthanized on day 3, and the right lung lobes were snap-frozen on dry ice and stored at −80°C for viral load analysis. The remaining mice (*n* = 4–5 per group) were euthanized at the end of the challenge observation period.

Prior to virus inoculation, vaccination, and blood collection, mice were anesthetized with 5% isoflurane at an oxygen flow rate of 2 L/min for approximately 3–5 min until a surgical plane of anesthesia was confirmed in accordance with AVMA Guidelines (2020). Inhalation was delivered using a precision vaporizer (EZ-7000 Anesthesia System). Mice were euthanized via carbon dioxide (CO_2_) inhalation using an automated system with a gradual chamber fill at a displacement rate of 30%–70% of the chamber volume per minute, followed by cervical dislocation as a secondary physical method to ensure death in accordance with the AVMA Guidelines for the Euthanasia of Animals (2020). After death was confirmed, tissues were collected from the mice.

### 2.4 Enzyme-linked immunosorbent assay

HA- and NA-specific serum antibody levels were determined by ELISA, as previously described (13). Briefly, Immulon 4HBX 96-well plates (Thermo Fisher, Waltham, MA, USA) were coated overnight at 4°C with 1 µg/mL of each COBRA recombinant HA (Y2, NG7, or BC17) or recombinant NA (N1-I or N2B) or WT HA (CA/09, HK/14, or PH/13) or NA (CA/09 or HK/14) in carbonate coating buffer (pH 9.4). Bovine serum albumin (BSA) (5 µg/mL) was used as a negative control. Following overnight incubation at 4°C, coating solution was removed, and plates were blocked for 90 min at 37°C with 200 µL/well of 1% BSA in PBST (PBS containing 0.05% Tween-20) (Thermo Fisher, Waltham, MA, USA) solution. After blocking, the buffer was decanted from the plates. Raw sera from each experimental group were pooled and serially diluted threefold in blocking buffer (starting at 1:250 for total IgG and 1:100 for IgG1 and IgG2a). A volume of 100 µL of diluted serum was added to each well of the ELISA plates. Each pooled serum sample was tested in duplicate. Plates were incubated for 2 h at 37°C in a humidified chamber and then washed five times with 150 µL of wash buffer (0.5 µL of Tween-20/mL of 1× PBS) and blotted dry on absorbent pads. Next, 100 µL of horseradish peroxidase (HRP)-conjugated secondary antibody diluted 1:4,000 in blocking buffer was added to each well. Three different secondary antibodies were used in separate assay plates: goat anti-mouse IgG-HRP (1 mg/mL) (Cat. no. 1030-05, RRID: AB_2619742, Southern Biotech, Birmingham, AL, USA), goat anti-mouse IgG1-HRP (1 mg/mL) (Cat. no. 1070-05, RRID: AB_2650509, Southern Biotech, Birmingham, AL, USA), and goat anti-mouse IgG2a-HRP (1 mg/mL) (Cat. no. 1083-05, RRID: AB_2794509, Southern Biotech, Birmingham, AL, USA). Plates were incubated for 90 min at 37°C and washed five times with wash buffer. After washing, plates were developed with 100 µL of 2,2′-azino-bis (3-ethylbenzothiazoline-6-sulfonic acid) (ABTS) substrate prepared in McIlvain’s buffer (pH 5.0) and incubated for 12 min at 37°C in a humidified chamber. The colorimetric reaction was stopped by adding 50 µL of 1% sodium dodecyl sulfate (SDS) (Thermo Fisher, Waltham, MA, USA) in dH_2_O. Optical density (O.D.) values were measured at 414 nm using a spectrophotometer (PowerWave XS, BioTek, Winooski, VT, USA). Background values were subtracted from each replicate, and the data were plotted in Prism against serum dilution. Total area under the curve (AUC) and standard error from the linear graphs were calculated and used for graphical representation.

### 2.5 Hemagglutination inhibition assay

HAI assay was performed to detect levels of anti-HA antibodies present in serum samples that inhibit attachment of the virus to red blood cells (RBCs), preventing cell hemagglutination (25). Serum antibody responses were evaluated against multiple influenza viruses, including H1N1 strains (BR/18, GD/19, and VC/19), H3N2 strains (SW/13, SA/19, HJ/19, and TS/20), and influenza B strains (BR/08, CO/17 and WA/19). Prior to the assay, serum samples were treated with receptor-destroying enzyme (RDE) (Denka Seiken Co., Tokyo, Japan) to remove nonspecific inhibitors of hemagglutination. Serum samples were incubated overnight at 37°C with RDE, followed by heat inactivation at 56°C for 45 min. PBS was then added to achieve a final serum dilution of 1:10.

RDE-treated serum samples were serially diluted twofold in PBS in 96-well V-bottom microtiter plates. An equal volume of virus diluted to 8 hemagglutination units (HAU)/50 µL was added to each well and incubated for 20 min (H1N1 and IBVs) or 30 min (H3N2 viruses). For H3N2 viruses, oseltamivir carboxylate (20 nM) was included in the virus mixture. Following incubation, 0.8% TRBCs (Lampire Biological Laboratories, Pipersville, PA, USA) were added for H1N1 and IBVs, and 0.8% GPRBCs were added for H3N2 viruses. The plates were incubated at room temperature (RT) for 30 min (H1N1 and influenza B) or 1 h (H3N2). Prior to use, RBCs were washed twice with PBS, stored at 4°C, and used within 24 h of preparation. HAI titers were determined as the reciprocal of the highest serum dilution that completely inhibited hemagglutination. Positive and negative serum controls were included on each plate. A seroprotective HAI antibody titer was defined as ≥1:40, and seroconversion was defined as a fourfold increase in HAI titer compared to baseline, according to WHO and European Committee for Medicinal Products guidelines for evaluating influenza vaccines (28).

### 2.6 HA/NA-specific antibody-secreting cells

Mouse spleens collected 9 days after final vaccination (day 37) from vaccinated pre-immune mice were homogenized into single-cell suspensions, resuspended in fetal bovine serum (FBS) (Thermo Fisher) + 10% dimethyl sulfoxide (DMSO) (Thermo Fisher), and stored in liquid nitrogen for future use. Upon thawing at 37°C, cells were washed and resuspended in complete B-cell medium (BCM). Individual samples (*n* = 5 mice) per vaccination group were assessed for H1 and H3 HA-specific antibody-secreting cells (ASCs). Mouse IgG T-cell enzyme-linked cytokine immunospot (ELISpot) (CTL, Shaker Heights, OH, USA) assay was performed following the manufacturer’s instructions. Briefly, ELISpot plates were first coated with COBRA Y2 (H1), NG7 (H3), BC17 (IBV), N1-I (N1), or N2B (N2) antigens or positive control (IgG capture antibody) and incubated overnight at 4°C. Plates were washed with PBS and splenocytes were incubated for 6 h at 37°C with 5% CO_2_. Plates were washed with PBS + 0.5% Tween-20 and incubated with tertiary antibody at RT in the dark for 1 h. Plates were washed with DI water, and substrate was added for 7 min at RT in the dark. After color development, plates were washed with tap water and left to dry overnight. Spots were scanned and counted using the ImmunoSpot S6 Ultimate Analyzer (CTL). Technical replicates for antibody secreting cell ELISpot assays were run in triplicate for each pooled sample.

### 2.7 IFN-γ/IL-17-secreting cell ELISpot

#### 2.7.1 Synthetic peptides

Overlapping 14–15-mer peptide arrays spanning the whole sequence of the HA and NA proteins from influenza A/H1N1, A/H3N2, and IBVs were obtained from the BEI Resources Repository (NIAID, NIH), including HA peptides from A/New York/18/2009 (NR-19245), A/Perth/16/2009 (NR-19266), and B/Malaysia/2506/2004 (NR-18967), and NA peptides from A/California/04/2009 (NR-18975) and A/Perth/16/2009 (NR-19267). For influenza A H1 and H3 HA proteins, 139 peptides were divided into six sub-pools (Head 1–4 and Stalk 1–2). For influenza A N1 and N2 NA proteins, 139 peptides were divided into five sub-pools (Head 1–4 and Stalk 1). For influenza B HA proteins, 95 peptides were divided into five sub-pools (Head 1–3 and Stalk 1–2). All peptide pools were used at a final concentration of 1 µg/mL per peptide in the assays.

#### 2.7.2 Enzyme-linked cytokine immunospot assay

**Figure 1:**
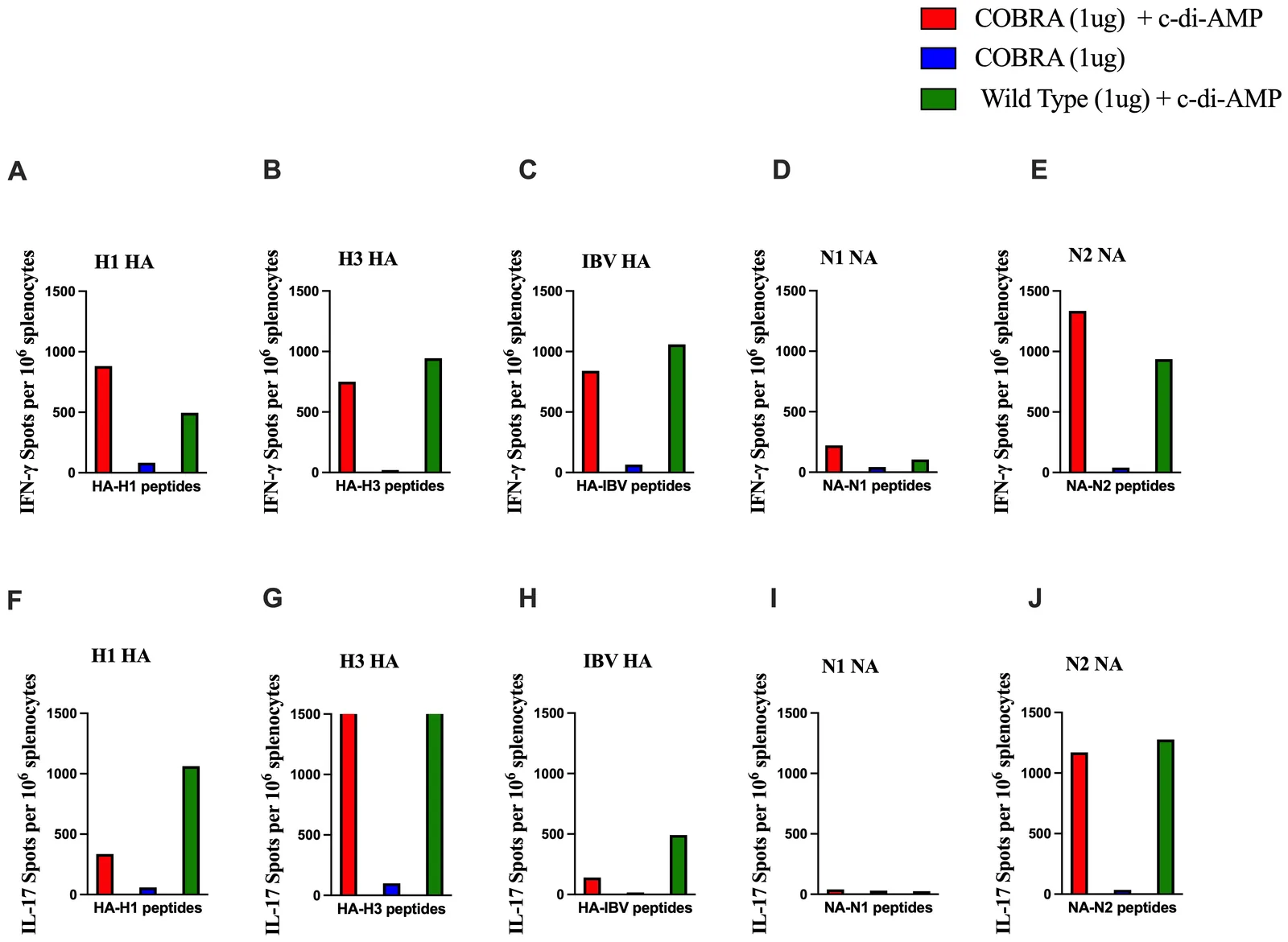
Quantification of influenza HA/NA/IBV-specific IFN-γ- and IL-17-secreting cells in pre-immune mice. Splenocytes collected on day 35 were isolated and pooled for each vaccine group. Cells (3×10^5^ per well) were stimulated *ex vivo* with specific 14–15-mer peptide pools spanning the full protein sequences of **(A, F)** A/New York/18/2009 [hemagglutinin (HA) H1 peptides], **(B, G)** A/Perth/16/2009 (HA H3 peptides), **(C, H)** B/Malaysia/2506/2004 (HA IBV peptides), **(D, I)** (A/California/04/2009) [neuraminidase (NA) N1 peptides], or **(E, J)** A/Perth/16/2009 (NA N2 peptides). The frequency of IFN-γ **(A–E)** - and IL-17 **(F–J)**-secreting cells was assessed by ELISpots assay. Descriptive data represent the number of spot-forming units (SFUs) per 10^6^ cells, for each pooled vaccine group.

ELISpot assays were performed using the Mouse ELISpot Kit (CTL, Shaker Heights, OH, USA; Cat. no. mIFN-γ/IL-17-1M/10). Ninety-six-well PVDF membrane plates were coated with purified murine anti-interferon-γ (IFN-γ) and anti-IL-17 antibodies and incubated overnight at 4°C. Spleens collected 7 days following the second vaccination (day 35) were homogenized, pooled per vaccination group (*n* = 2 per group), and assessed with IFN-γ-secreting (**Figures 1A–E**) and IL-17-secreting cells (**Figures 1F–J**). Splenocytes from immunized or mock-treated DBA/2J mice were harvested under sterile conditions using complete RPMI 1640 medium (Thermo Fisher, Waltham, MA, USA) supplemented with 1% fresh L-glutamine and processed using the gentleMACS Octo Dissociator (Miltenyi Biotec, MD, USA). After washing the plates, peptide pools diluted in CTL-Test Medium were added to designated wells at a 2× final concentration of 2 µg/mL per peptide, followed by 100 µL of splenocyte suspension (3 × 10^5^ cells/well). Each assay included an unstimulated control (cells in medium only) and a positive control [cells stimulated with PMA (50 ng/well, Fisher Scientific, Waltham, MA, USA) and ionomycin (500 ng/well, Sigma-Aldrich, St. Louis, MO, USA)]. Plates were incubated for 24 h at 37°C with 5% CO_2_, washed with PBS containing 0.1% Tween-20, and incubated with biotinylated anti-IFN-γ and anti-IL-17 detection antibodies for 2 h at RT. After washing, plates were incubated with FITC-HRP and Streptavidin-AP for 1 h, washed again, and developed using Blue and Red Developer Substrate Solutions (CTL, Shaker Heights, OH, USA). Spots were enumerated using a CTL ImmunoSpot^®^ reader and analyzed with ImmunoSpot^®^ Version 7 software. Each spot corresponds to an individual cytokine-secreting cell. Background responses from negative control wells were subtracted to normalize the data across all experimental groups.

### 2.8 Influenza viral plaque assay

Viral titers in mouse lungs were determined using a plaque assay. MDCK cells (1 × 10^6^ cells/well) were seeded into six-well plates 1 day prior to infection and maintained in complete Dulbecco’s Modified Eagle Medium (DMEM, Thermo Fisher, Waltham, MA, USA) supplemented with 10% FBS, 100 U/mL penicillin, 100 µg/mL streptomycin, and 25 mM HEPES. Collected mouse lungs were weighed, thawed on ice, and homogenized in 1 mL of DMEM. Homogenates were centrifuged at 2,000 rpm for 5 min to remove tissue debris, and the supernatant was serially diluted 10-fold in DMEM containing 1% penicillin–streptomycin. MDCK cells at ~90% confluency were washed three times with DMEM + P/S and infected with 100 µL of each dilution of lung homogenate supernatant. After 1 h of adsorption at RT with intermittent agitation, cells were washed twice with DMEM + P/S and overlaid with 2 mL of a 1.6% agarose and 2× MEM overlay (50:50 v/v) supplemented with 40 mM HEPES, 2× L-glutamine, 50 mg/mL gentamicin, 17.86 mM NaHCO_3_, 1 µg/mL TPCK-trypsin, 200 U/mL penicillin, and 200 µg/mL streptomycin. Plates were incubated at 37°C with 5% CO_2_ for 72 h to allow plaque formation. Overlays were removed, and cells were fixed with 10% neutral buffered formalin for 10 min, stained with 1% crystal violet for 10 min, and rinsed thoroughly with tap water five times. Plates were air-dried for 24 h, and viral plaques were enumerated. Lung viral titers were calculated as PFU per gram of lung tissue.

### 2.9 Statistical analysis

All data are presented as mean ± standard error of the mean (SEM). One-way analysis of variance (ANOVA) with multiple comparisons was used to analyze influenza strain-specific HAI titers and lung viral titers between vaccination groups. Differences in weight-loss curves were analyzed using a nonparametric Kruskal–Wallis one-way ANOVA with Dunn’s multiple comparisons. HAI titers were transformed to the log_2_ scale to standardize the wide range of antibody responses. Statistical analyses were performed using GraphPad Prism software version 10.2.1 (GraphPad Software, San Diego, CA, USA). A *p*-value < 0.05 was considered statistically significant (*p < 0.05, **p < 0.01, ***p < 0.001, ****p < 0.0001).

## 3 Results

### 3.1 Pentavalent COBRA rHA and rNA vaccination adjuvanted with c-di-AMP induces systemic influenza-specific IgG titers

**Figure 2:**
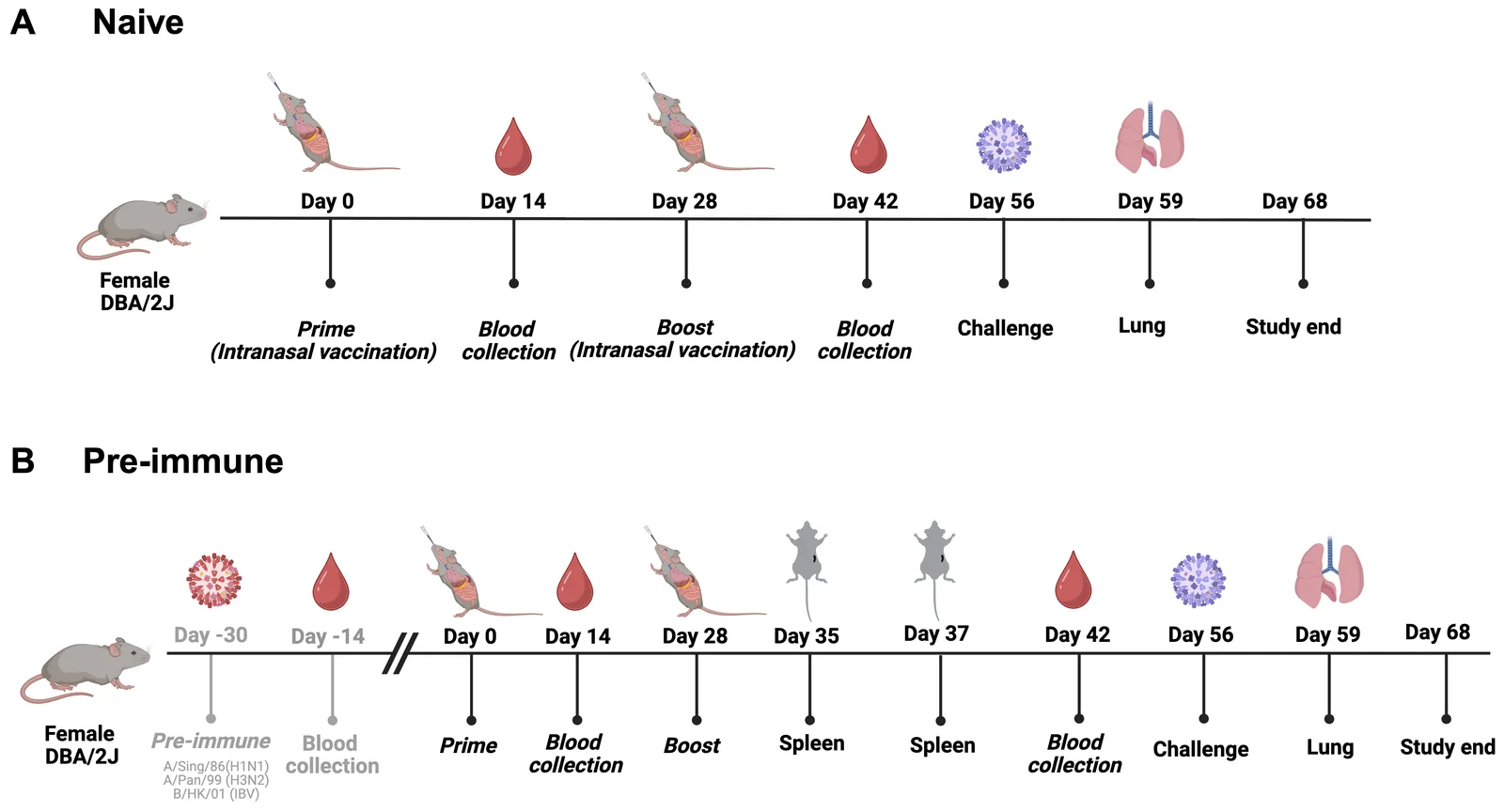
Schematic of experimental timeline. DBA/2J (6–8 weeks old) female mice were randomly divided into 18 groups. **(A)** Nine groups of immunologically naïve mice (*n* = 23–24 mice/group) were vaccinated intranasally, in a prime-boost regimen with pentavalent formulation of each COBRA HA (Y2, NG7, and BC17) and COBRA NA (N1-I and N2-B) or WT HA (CA/09, HK/14, and PH/13) and WT NA (CA/09 and HK/14) proteins. The pentavalent combination of vaccines was administered at two different concentrations (0.1 or 1 µg), either with or without adjuvant on days 0 and 28. As control, one group was inoculated with PBS. Blood was collected from the mice on days 14 and 42. On day 56, each group of mice were intranasally challenged with BR/18 (H1N1), SW/13 (H3N2), or WA/19 (IBV) (*n* = 7–8 mice per viral subtype). Three days post-infection, lungs were harvested from a subset of mice (*n* = 3 per viral subtype), and the remainder of the mice (*n* = 4–5 per group) were monitored for 12 days post-infection. **(B)** Mice were infected intranasally on day −30 with the historical viruses: H1N1 SG/86 (5×10^5^ PFU/mouse), H3N2 PN/99 (5×10^5^ PFU/mouse), and B/HK/01 (1×10² PFU/mouse) for a total of 50 μL dose per mouse. All pre-immune mice (*n* = 234) were randomly divided into nine groups and intranasally vaccinated with pentavalent formulation of each COBRA HA (Y2, NG7, and BC17) and COBRA NA (N1-I and N2-B) or WT HA (CA/09, HK/14, and PH/1) and WT NA (CA/09 and HK/14) proteins. The pentavalent combination of vaccines was administered at two different concentrations (0.1 or 1 µg), either with or without adjuvant on days 0 and 28. As control, one group was inoculated with PBS. Pre-immune mice were bled on days −14, 14, and 42. Spleens were harvested on days 35 (*n* = 2/group) and 37 (*n* = 5/group) from three selected groups of mice that received 1 µg of pentavalent formulation. On day 56, mice were challenged with BR/18 (H1N1), SW/13 (H3N2), or WA/19 (IBV) (*n* = 7–8 mice per viral subtype). Three days post-infection, lungs were harvested from a subset of mice (*n* = 3 per viral subtype), and weights were recorded (*n* = 4–5 per mice group) for 12 days after challenge. Created in BioRender. Caetano, C. (2026) https://BioRender.com/72as9lw.

**Figure 3:**
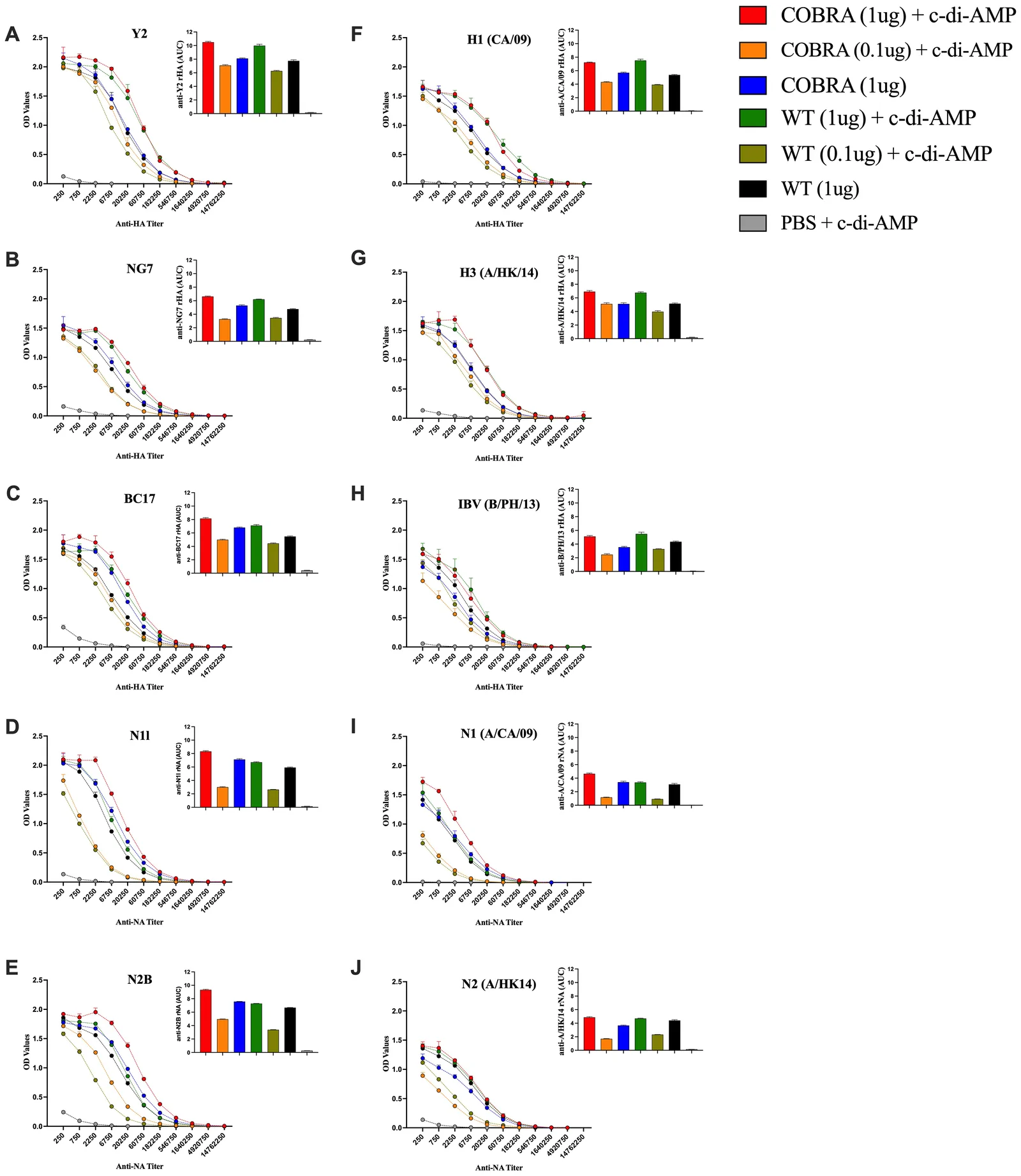
Anti-HA/NA IgG in intranasally vaccinated pre-immune mice. Serum collected from pre-immune DBA/2J mice 14 days after the second vaccination were pooled for each group (5 mice/group) and assessed for COBRA antigens **(A)** H1-specific total IgG against Y2 rHA, **(B)** H3-specific total IgG against NG7, **(C)** IBV-specific total IgG against BC17, **(D)** N1-specific total IgG against N1I rNA, or **(E)** N2-specific total IgG against N2B rNA. Anti-HA/NA IgG binding was also tested against the wild-type antigens **(F)** H1 (A/CA/09) rHA, **(G)** H3 (A/HK/14) rHA, **(H)** IBV (B/PH/13) rHA, **(I)** N1 (A/CA/09) rNA, and **(J)** N2 (A/HK/14) rNA. Replicates for each pooled sample, after background subtraction, were plotted as optical density at 450 nm (OD_450_) values against serial dilutions using Prism. Data points represent mean OD values of replicates. Total area under the curve (AUC) and standard error from the linear graphs were calculated for quantitative comparison in bar graphs. The legend indicates antigen dose, presence of c-di-AMP adjuvant, and the PBS control group.

Immunologically naïve (**Figure 2A**) and pre-immune (**Figure 2B**) DBA/2J mice were randomly assigned to nine groups and vaccinated twice intranasally in a prime-boost regimen. Mice received different concentrations of pentavalent formulation containing COBRA recombinant HA (Y2, NG7, or BC17), recombinant NA (N1-I or N2B), or WT HA (CA/09, HK/14, or PH/13) or NA (CA/09 or HK/14) antigens, adjuvanted with a c-di-AMP emulsion or PBS (**Table 1**). To evaluate the breadth of the humoral immune response elicited by distinct immunization strategies, virus-specific IgG responses were measured in sera collected from pre-immune mice after the second vaccination. Antibody responses were assessed against a diverse panel of COBRA and WT influenza HA (H1, H3, and IBV) and NA (N1 and N2) proteins (**Figure 3**). All vaccinated mice had anti-HA and anti-NA antibodies that bound to rHA/NA proteins representing the H1, H3, B HA, and NA proteins in the vaccine. Across all tested COBRA antigens—Y2, NG7, BC17, N1-I, and N2B (**Figures 3A–E**), mice vaccinated with 1 μg of the pentavalent COBRA formulation adjuvanted with c-di-AMP consistently had the higher IgG titers and increased AUC values compared to mice vaccinated with WT or unadjuvanted COBRA formulations. In general, mice vaccinated with WT antigens had lower IgG titers, and the addition of c-di-AMP enhanced antibody titers (**Figures 3F–J**). There was a dose-dependent effect with mice vaccinated with the high dose of 1 µg of HA or NA eliciting 2- to 4-fold higher IgG titers compared to mice vaccinated with a low dose of 0.1 µg of each protein plus adjuvant. Mice vaccinated with pentavalent COBRA and WT antigens had primarily IgG1 antibodies (IgG1:IgG2a ratios > 1) following vaccination. Mice that received the unadjuvanted vaccine had the highest IgG1:IgG2a ratios (often >4.5) (**Supplementary Figure 1A**). Notably, the inclusion of c-di-AMP promoted a more balanced IgG1:IgG2a ratio compared to mice administered unadjuvanted vaccine.

**Table 1:** Experimental groups and immunization formulations.

Groups	Vaccine	Dose	Adjuvant
1	COBRA pentavalent	1 µg/protein	c-di-AMP
2	COBRA pentavalent	0.1 µg/protein	c-di-AMP
3	COBRA pentavalent	1 µg/protein	None
4	COBRA pentavalent	0.1 µg/protein	None
5	WT pentavalent	1 µg/protein	c-di-AMP
6	WT pentavalent	0.1 µg/protein	c-di-AMP
7	WT pentavalent	1 µg/protein	None
8	WT pentavalent	0.1 µg/protein	None
9	Mock	0 µg/protein	c-di-AMP

Computationally optimized broadly reactive antigen (COBRA) methodology was used to generate hemagglutinin (HA) and neuraminidase (NA) immunogens for the pentavalent influenza vaccine. COBRA recombinant proteins included Y2 HA (H1), NG7 HA (H3), BC17 HA (IBV), N1I NA (N1), and N2B NA (N2), and wild-type (WT) included CA/09 HA (H1), HK/14 HA (H3), PH/13 HA (IBV), CA/09 NA (N1), and HK/14 NA (N2). DBA/2J female naïve and pre-immune mice were divided into nine experimental groups each and immunized intranasally with pentavalent formulations containing COBRA or WT HA/NA proteins at 1 or 0.1 μg per mouse, administered either with or without adjuvant c-di-AMP adjuvant. A PBS + c-di-AMP group served as the mock control.

### 3.2 Pentavalent HA and NA vaccination adjuvanted with c-di-AMP elicits protective H1N1, H3N2, and IBV HAI titers

**Figure 4:**
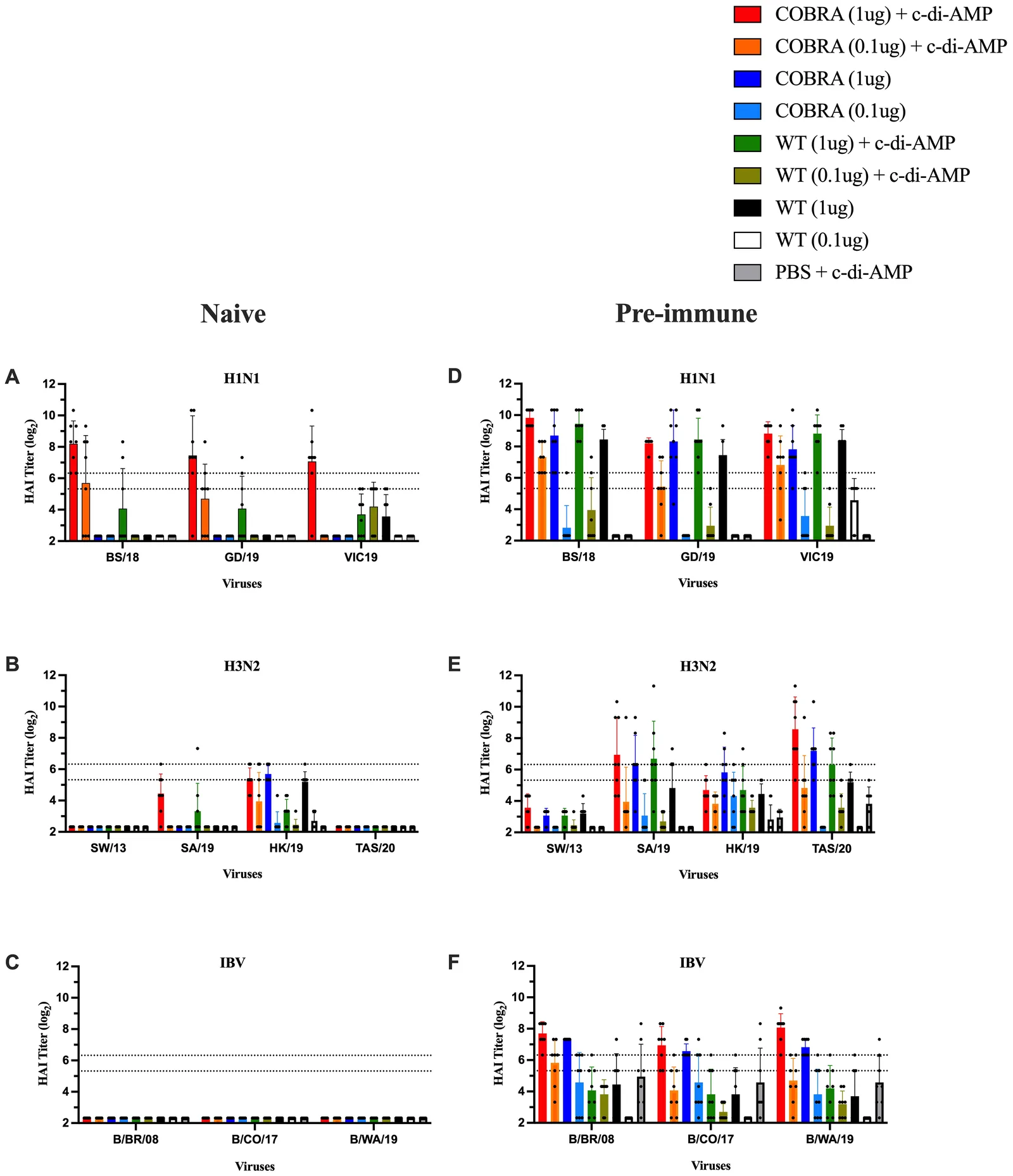
Serum hemagglutination inhibition activity in naïve and pre-immune mice following prime-boost vaccinations. Hemagglutination inhibition (HAI) titers of mice serum antibodies against a panel of H1N1, H3N2, and influenza B virus strains after immunization with the different pentavalent vaccine formulations described in **Table 1**. Sera from **(A–C)** naïve and **(D–F)** pre-immune mice were collected 14 days after the second vaccination for HAI assay against a panel of **(A, D)** H1N1, **(B, E)** H3N2, and **(C, F)** influenza B viruses. Strains tested and listed along the *x*-axis include H1N1 (BR/18, GD/19, and VC/19); H3N2 (SW/13, SA/19, HK/19, and TAS/20); and IBV (B/BR/08, B/CO/17, and B/WA/19). Mean Log_2_ HAI titers are indicated by the *y*-axis with error bars depicting standard deviation. Dotted horizontal lines represent the thresholds for protective HAI titers, in which the top line indicates a 1:80 HAI titer and the bottom line indicates a 1:40 HAI titer. Bars represent the mean titer ± SEM, with individual mice shown as black dots. Statistical significance was determined by Kruskal–Wallis ANOVA with Dunn’s multiple comparison test to determine statistical significance between vaccine groups (*p < 0.05, **p < 0.01, ***p < 0.001, ****p < 0.0001).

Following the analysis of antibody responses, the antibody magnitude and breadth elicited by different vaccine formulations were assessed in a HAI activity against panels of H1N1, H3N2, and IBVs (**Figure 4**). Both naïve and pre-immune mice vaccinated with the high-dose pentavalent COBRA HA/NA proteins adjuvanted with c-di-AMP had seroprotective HAI titers (≥1:80) against three H1N1 influenza strains (**Figures 4A, D**). In immunologically naive mice (**Figures 4A–C**), high-dose COBRA HA/NA proteins plus c-di-AMP (red) elicited significantly higher HAI titers (≥1:80) against H1N1 strains BR/18, GD/19, and VC/19 compared to most other vaccinated mouse groups (*p* < 0.0001) (**Figure 4A**), whereas non-adjuvanted or WT controls had low to undetectable HAI titers.

There were low to no detectable HAI titers (≥1:40) in vaccinated naïve mice following vaccination (**Figure 4B**). However, pre-immune mice vaccinated with a high dose of vaccine had higher HAI titers than naïve mice against two of the four viruses in the panel (SA/19 and TS/20) (**Figure 4E**). Mice vaccinated with a high dose of vaccine had significantly higher HAI titers, in general, than mice vaccinated with a low dose of vaccine, as well as mock-vaccinated mice (*p* < 0.0001) (**Supplementary Tables 1**, **2**). Vaccinated naïve mice showed low HAI activity for H3N2 viruses following vaccination (**Figure 4B**). Slightly higher HAI titers against SA/19 and HK/19 were observed in mice that received the high-dose vaccine formulation with adjuvant (**Figure 4B**). There were no detectable HAI titers against any of the IBV strains (**Figure 4C**). In contrast, pre-immune mice vaccinated with a high dose of vaccine with or without adjuvant had an average seroprotective HAI titer ≥ 1:80 against the H1N1 influenza virus panel (**Figure 4D**) and average HAI titers ≥ 1:40 against three of the four viruses in the H3N2 panel (**Figure 4E**). Mice vaccinated with low-dose vaccines plus c-di-AMP enhanced seroprotective HAI titers (orange) against H1N1 influenza viruses (**Figure 4D**) and IBV (BR/08) (**Figure 4E**). Particularly for IBV (**Figure 4F**), mice vaccinated with high-dose COBRA proteins plus c-di-AMP had significantly higher titers against the panel of IBV strains compared to mice vaccinated with high-dose WT proteins plus adjuvant (*p* < 0.05) (**Supplementary Table 2**). Mock-vaccinated mice, regardless of whether they were naïve or had pre-existing influenza immunity, had no detectable protective HAI titers.

### 3.3 Pentavalent rHA/rNA vaccines elicit robust antibody-secreting cells in pre-immune mice

**Figure 5:**
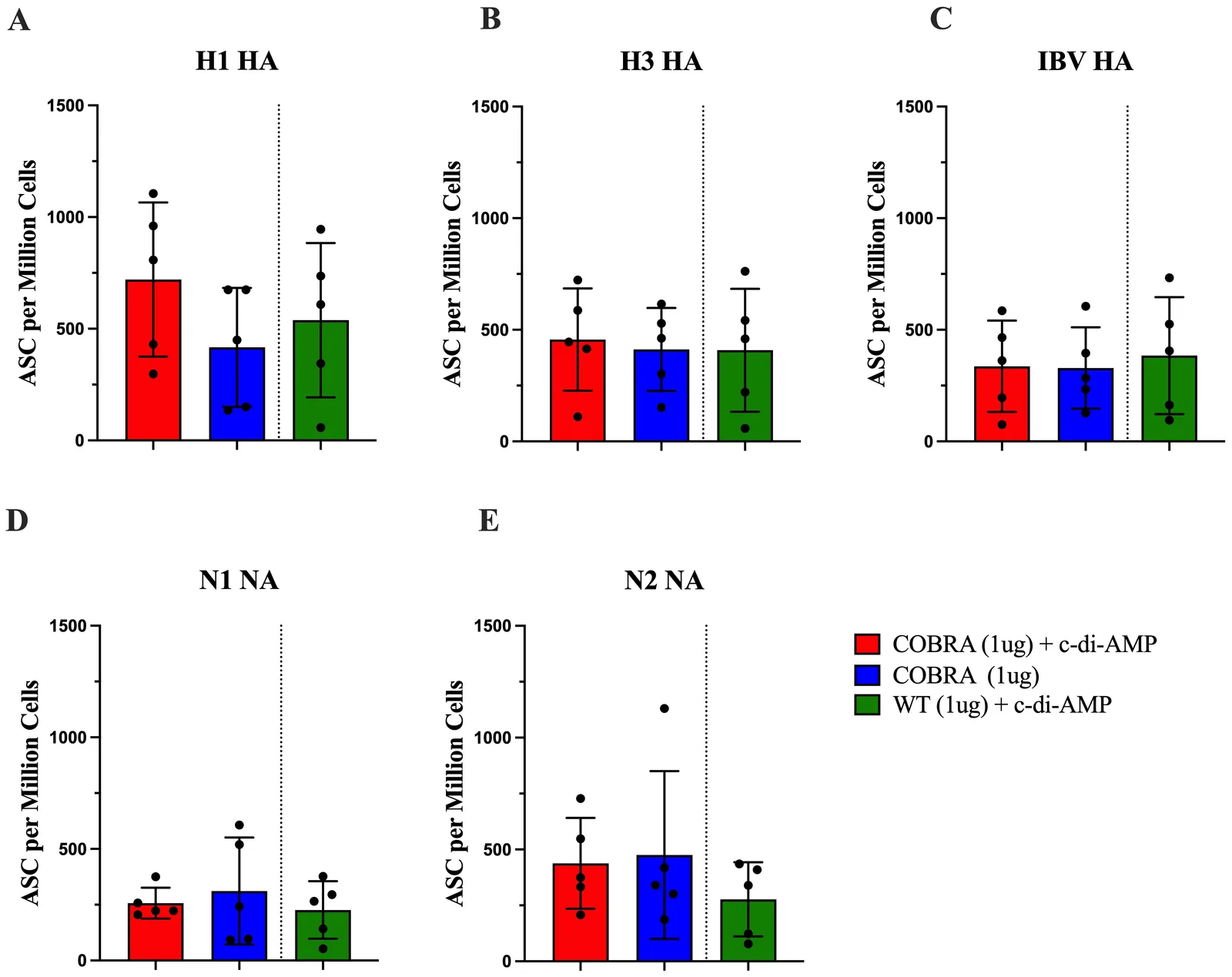
Quantification of influenza HA- and NA-specific antibody-secreting cells (ASCs) in pre-immune mice. DBA/2J pre-immune mice were vaccinated intranasally in a prime-boost regimen with pentavalent formulation of COBRA or wild-type hemagglutinin (HA) and neuraminidase (NA) vaccines using c-di-AMP as adjuvant. Splenocytes were collected 9 days after the final vaccination for ELISpots assay, and cells were probed for antigen-specific ASCs against vaccine components **(A)** H1 HA, **(B)** H3 HA, **(C)** IBV-HA, **(D)** N1 NA, and **(E)** N2 NA. The *y*-axis represents the number of ASCs per one million input cells collected from each mouse, and the vaccine groups are listed on the figure legends.

Mouse splenocytes were used to determine the number of antigen-specific ASCs in pre-immunized mice that received a high dose (1 μg) of vaccine. Spleens were homogenized and assessed for HA (H1, H3, and IBV)- and NA (N1 and N2)-specific ASCs. Overall, all mice vaccinated with high-dose COBRA or WT protein formulations had robust numbers of ASCs against all the HA and NA antigens in the vaccine that ranged from ~250 to 1,000 ASCs per million splenocytes, regardless of whether the COBRA vaccine was mixed with or without adjuvant (**Figures 5A–E**). For H1 HA, mice vaccinated with the adjuvanted COBRA rHA/NA vaccine had a higher number of H1-specific ASCs (~700–1,000 ASCs per million cells) compared to mice administered non-adjuvanted COBRA HA/NA vaccines (**Figure 5A**). This corresponded to an ~1.5-fold increase in the number of ASCs. In contrast, the number of H3 and IBV HA-specific ASCs was comparable across all vaccinated groups, with mean values clustering between ~350 and 500 ASCs per million cells (**Figures 5B, C**). NA-specific ASC responses were more modest in magnitude. N1-specific responses averaged ~250–300 ASCs per million cells (**Figure 5D**), while N2-specific responses averaged ~500 ASCs per million cells (**Figure 5E**). For both NA antigens, the number of anti-NA ASCs was similar between adjuvanted and non-adjuvanted groups.

### 3.4 Adjuvanted HA/NA vaccines elicited a greater frequency of IFN-γ- and IL-17-secreting antigen-specific splenocytes than unadjuvanted vaccines

To evaluate cellular immune responses induced by intranasal vaccination, influenza-specific T-cell responses were evaluated by ELISpot assay in pre-immune mice. Seven days after the booster vaccination, splenocytes were harvested from vaccinated mice and stimulated with peptide pools representing from HA (H1, H3, and IBV) and NA (N1 and N2) for *ex vivo* quantification of cytokine-secreting T cells. Responses were quantified via IFN-γ- and IL-17-secreting T cells. Descriptively, mice vaccinated with c-di-AMP adjuvanted vaccines had greater frequency of IFN-γ- and IL-17-specific cytokine-secreting cells against all antigens in the vaccine (**Figures 1A–J**). Graphic representation of the spots upon stimulation with HA/NA head and stem peptide pools is depicted in **Supplementary Figure 2**. Assessment of IFN-γ-secreting cells revealed that the mice vaccinated with HA/NA proteins plus adjuvant had robust T-cell responses against multiple HA and NA proteins representing influenza subtypes (**Figures 1A–E**).

Stimulating splenocytes with H1 HA peptides resulted in 880 IFN-γ spot-forming units (SFU) per 10^6^ splenocytes in the COBRA + c-di-AMP vaccinated mice compared to ~500 SFU in the WT plus c-di-AMP vaccinated mice (**Figure 1A**; **Supplementary Figure 2**). For HA-H3N2, mice vaccinated with COBRA plus c-di-AMP had ~750 SFU following stimulation with H3 HA peptides, while ~950 IFN-γ-secreting spots were detected in splenocyte stimulation with peptides from mice vaccinated with adjuvanted WT formulation (**Figure 1B**; **Supplementary Figure 2**). Responses to IBV HA were comparable between the COBRA plus c-di-AMP (~800 SFU) and WT plus c-di-AMP (~1,050 SFU) groups (**Figure 1C**). While the responses to N1 NA remained relatively low (<250 SFU) in all vaccinated mice (**Figure 1D**), the COBRA plus c-di-AMP vaccine appeared to have a notably higher N2 NA-specific response (~1,350 SFU) compared to WT plus c-di-AMP (~950 SFU) (**Figure 1E**).

Consistent with the IFN-γ data, mice vaccinated with unadjuvanted COBRA HA/NA proteins had a low number of IL-17-secreting T cells following stimulation with HA- and NA-specific peptides (**Figures 1F–J**; **Supplementary Figure 2**). Mice vaccinated with COBRA plus c-di-AMP had a high number of IL-17-secreting cells following stimulation with H3 HA peptides (**Figure 1G**) and N2 NA peptide pools (**Figure 1J**). However, for HA-H1N1 (**Figure 1F**) and HA-IBV (**Figure 1H**) pools, mice vaccinated with the COBRA plus c-di-AMP vaccine had few IL-17-secreting T cells compared to mice vaccinated with WT HA/NA proteins plus c-di-AMP. Similar to the IFN-γ responses, mice vaccinated with N1 NA had few IL-17-secreting splenocytes following vaccination (**Figure 1I**). There were a high number of IL-17-secreting T cells following N2 NA peptides in mice vaccinated with either the COBRA HA/NA proteins plus c-di-AMP (~1,150 SFU) or WT HA/NA proteins plus c-di-AMP (~1,250 SFU) (**Figure 1J**).

### 3.5 COBRA-based vaccination adjuvanted with c-di-AMP protects mice against lethal influenza virus challenge

**Figure 6:**
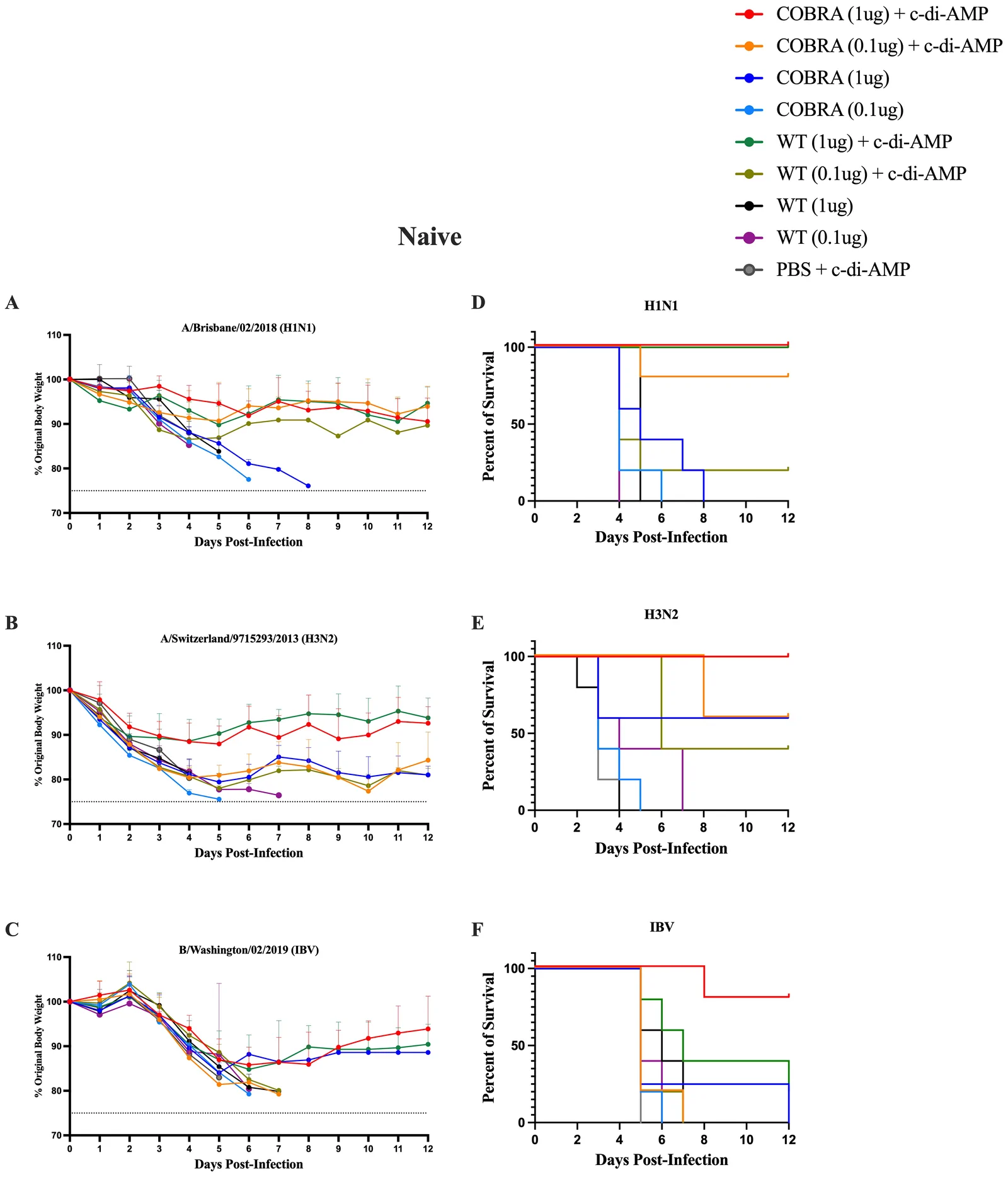
Percent of original mouse weight and percent survival following influenza virus challenge in naïve mice. DBA/2J naïve mice were vaccinated intranasally two times at 4-week intervals as described in **Figure 2A**. Vaccine groups were pentavalent COBRA or WT HA and NA formulated with or without adjuvant as described in **Table 1**. Weight and survival of mice were recorded daily after challenge with **(A, D)** 10^4^ PFU of BR/18 (H1N1), **(B, E)** 10^6^ PFU of SW/13 (H3N2), and **(C, F)** 10^3^ PFU of WA/19 (IBV) viruses. Weight loss **(A–C)** and percent survival **(D–F)** are shown over a 12-day period. Statistical significance was assessed using Kruskal–Wallis test (one-way ANOVA) followed by Dunn’s multiple comparisons test to evaluate differences between vaccine groups at each day post-infection (*p < 0.05, **p < 0.01, ***p < 0.001, ****p < 0.0001).

**Figure 7:**
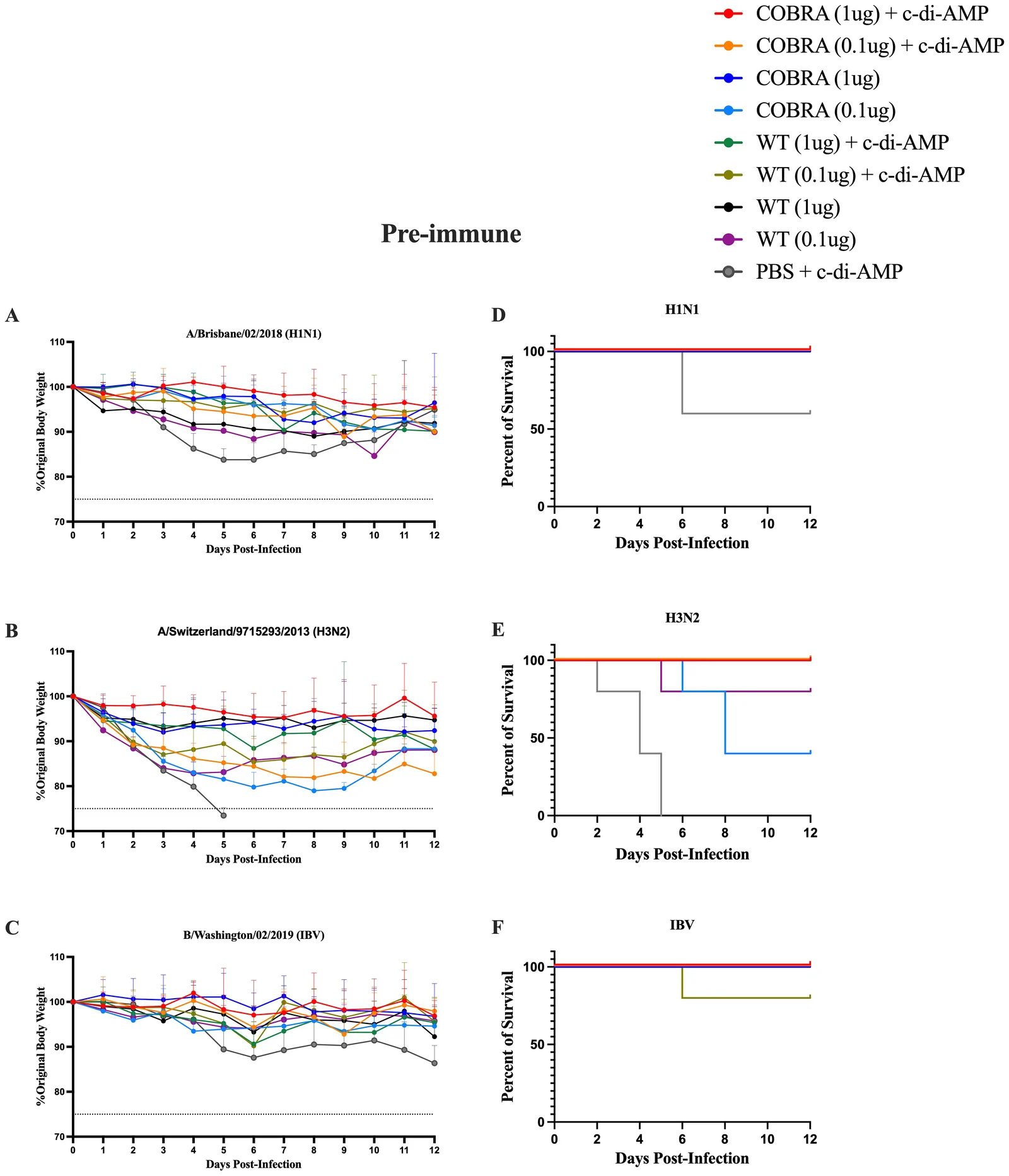
Percent of original mouse weight and percent survival following influenza virus challenge in pre-immune mice. DBA/2J pre-immune mice were vaccinated intranasally two times at 4-week intervals as described in **Figure 2B**. Vaccine groups were pentavalent COBRA or WT HA and NA formulated with or without adjuvant as described in **Table 1**. Weight and survival of mice were recorded daily after challenge with **(A, D)** 10^4^ PFU of BR/18 (H1N1), **(B, E)** 10^6^ PFU of SW/13 (H3N2), and **(C, F)** 10^3^ PFU of WA/19 (IBV) viruses. Weight loss **(A–C)** and percent survival **(D–F)** are shown over a 12-day period. Statistical significance was assessed using Kruskal–Wallis test (one-way ANOVA) followed by Dunn’s multiple comparisons test to evaluate differences between vaccine groups at each day post-infection (*p < 0.05, **p < 0.01, ***p < 0.001, ****p < 0.0001).

**Figure 8:**
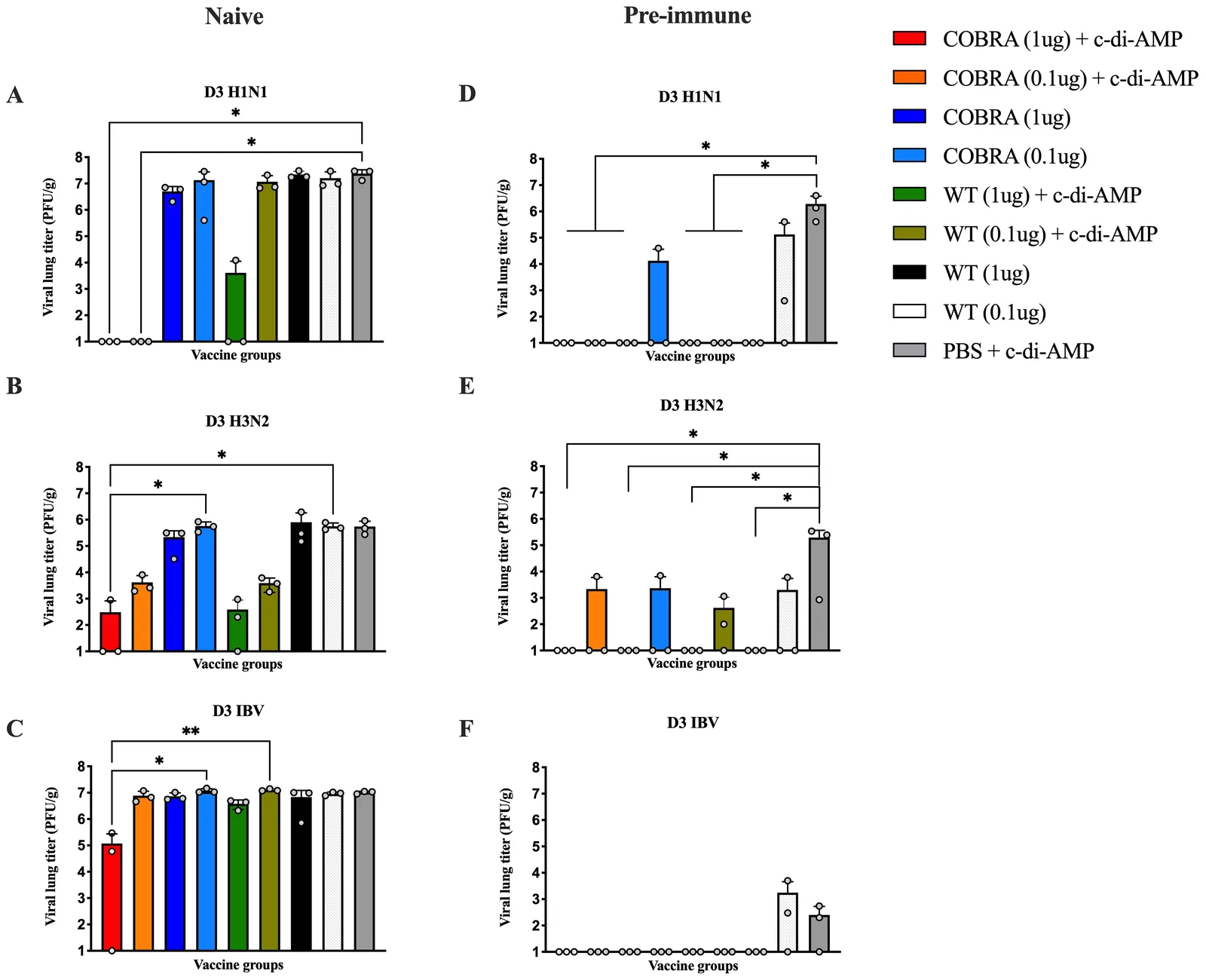
Viral titers in lung tissue after challenge in naïve and pre-immune mice. Viral replication was assessed by plaque assay in lung homogenates collected on day 3 post-challenge (*n* = 3 mice per viral subtype). Naïve **(A–C)** and pre-immune **(D–F)** mice were intranasally vaccinated with pentavalent COBRA or WT HA and NA formulated with or without adjuvant. Four weeks after final vaccination, mice were intranasally infected with lethal doses of **(A, D)** BR/18 (H1N1), **(B, E)** SW/13 (H3N2), or **(C, F)** WA/19 (IBV). Viral titers in lung tissues are presented as PFU/g of tissue shown on the *y*-axis. Each dot represents one individual mouse. Statistical significance was assessed using Kruskal–Wallis test (one-way ANOVA) followed by Dunn’s multiple comparisons test to evaluate differences between vaccine groups at each day post-infection (*p < 0.05, **p < 0.01, ***p < 0.001, ****p < 0.0001).

Both naïve and pre-immune vaccinated mice were challenged 28 days after the booster vaccination (day 56) with a lethal dose of either H1N1 (BR18), H3N2 (SW/13), or IBV (WA/19). Following challenge, all mice were evaluated for morbidity and mortality for 12 days post-infection (**Figures 6**, **7**; **Table 2**). On day 3 post-infection, lungs were harvested and assessed for virus replication (**Figure 8**).

**Table 2:** Percent survival of vaccinated mice following viral challenge.

A			
Naïve cohort			
Group	H1N1 survival (%)	H3N2 survival (%)	IBV survival (%)
COBRA (1 μg) + c-di-AMP	100%	100%	80%
COBRA (0.1 μg) + c-di-AMP	80%	60%	0%
COBRA (1 μg)	0%	60%	20%
COBRA (0.1 μg)	0%	0%	0%
WT (1 μg) + c-di-AMP	100%	100%	40%
WT (0.1 μg) + c-di-AMP	20%	40%	0%
WT (1 μg)	0%	0%	0%
WT (0.1 μg)	0%	0%	0%
PBS + c-di-AMP	0%	0%	0%

B			
Pre-immune cohort			
Group	H1N1 survival (%)	H3N2 survival (%)	IBV survival (%)
COBRA (1 μg) + c-di-AMP	100%	100%	100%
COBRA (0.1 μg) + c-di-AMP	100%	100%	100%
COBRA (1 μg)	100%	100%	100%
COBRA (0.1 μg)	100%	40%	100%
WT (1 μg) + c-di-AMP	100%	100%	100%
WT (0.1 μg) + c-di-AMP	100%	100%	80%
WT (1 μg)	100%	100%	100%
WT (0.1 μg)	100%	80%	100%
PBS + c-di-AMP	60%	0%	100%

Mice were challenged on day 56 with BR/18 (H1N1), SW/13 (H3N2), or WA/19 (IBV) after immunization with varying doses of COBRA or wild-type antigens, with or without c-di-AMP adjuvant. Summary of the final survival percentages for mice in the **(A)** naïve and **(B)** pre-immune cohorts from day 1 to day 12 post-challenge.

Following challenge with BR/18 (**Figure 6A**), mock-vaccinated naïve mice (gray) rapidly lost weight and reached humane endpoint on day 4 post-infection. Naïve mice vaccinated with a high dose of COBRA HA/NA plus adjuvant (red), a low dose of COBRA HA/NA plus adjuvant (orange), or a high dose of WT HA/NA plus adjutant (green) had, on average, less than 7% weight loss with no detectable clinical signs, and most mice survived challenge (**Figures 6A, D**). In contrast, naïve mice vaccinated with low-dose WT HA/NA proteins plus adjuvant lost ~13% weight by day 5 post-challenge with H1N1 (**Figure 6A**) with 20% survival (**Figure 6D**, **Table 2A**; **Supplementary Table 3A**). Naïve mice vaccinated with both adjuvanted high- or low-dose COBRA vaccine had no detectable lung viral titers on day 3 post-H1N1 challenge (**Figure 8A**). Mice vaccinated with a high dose of WT HA/NA proteins plus adjuvant also had, on average, ~3.5 log_10_ PFU/g viral lung titers. Mice vaccinated with any other formulation of COBRA or WT HA/NA vaccines had similar high viral lung titers (>6.5 log_10_ PFU/g) to mock-vaccinated mice (**Figure 8A**).

Mock-vaccinated mice that were challenged with the H3N2 influenza virus, SW/13, lost, on average, ~25% of their original body weight by days 3–4 post-challenge (**Figure 6B**) with no mice surviving challenge (**Figure 6E**; **Table 2A**). Mice vaccinated with high-dose COBRA or WT HA/NA vaccines with c-di-AMP lost, on average, ~10% of their original weight by day 5 post-challenge and slowly recovered (**Figure 6B**; **Supplementary Table 3B**) with all mice surviving challenge (**Figure 6E**, **Table 2A**). In contrast, naïve mice vaccinated with unadjuvanted low-dose COBRA and WT HA/NA vaccine lost ~25% of their original weight by day 5 post-H3N2 challenge (**Figure 6B**) with 0% surviving after challenge (**Figure 6E**, **Table 2A**). Mice vaccinated with any of the other formulations lost, on average, ~20% of their original body weight with various amounts of survival per group. Naïve mice vaccinated with either adjuvanted high-dose COBRA HA/NA proteins or adjuvanted high-dose WT HA/NA proteins had >10^3^ PFU/g viral lung titers on day 3 post-challenge with H3N2, while mice vaccinated with their low-dose counterparts had viral lung titers >10^4^ PFU/g (**Figure 8B**). Mice vaccinated with unadjuvanted vaccines or mock vaccinated had viral lung titers between 5.5×10^5^ and 1×10^6^ PFU/g (**Figure 8B**).

Naïve mice vaccinated with low-dose COBRA HA/HA or WT HA/NA vaccines or mock vaccinated and challenged with the WA/19 IBV lost ~20% of their body weight by days 6–7 post-challenge (**Figure 6C**) with no mice surviving challenge (**Figure 6F**; **Table 2A**). Mice vaccinated with high dose of the COBRA HA/NA proteins, with or without adjuvant, or WT HA/NA proteins with adjuvant lost, on average, ~13% of their original body weight by day 7 post-challenge. Mice vaccinated with COBRA HA/NA proteins plus adjuvant had 80% of mice survive to day 12 post-infection, but only 20% of mice survived in the other two groups (**Figure 6F**; **Table 2A**). Mock-vaccinated mice had an average of 1×10^7^ PFU/g viral lung titers on day 3 post-IBV challenge, which was similar to all other vaccinated mice, except for mice vaccinated with high-dose COBRA HA/NA proteins plus adjuvant, which had an average of two lower viral lung titer at 1×10^5^ PFU/g (**Figure 8C**).

In contrast to naïve vaccinated mice, a set of mice with pre-existing influenza immune responses was vaccinated and then challenged with the same influenza viruses (**Figure 7**). Pre-immune mock-vaccinated mice lost, on average, ~15% of their original body weight by day 5 following infection with the BR/18 H1N1 influenza virus and then regained weight over the next 10 days (**Figure 7A**) with 60% of the mice surviving challenge (**Figure 7D**; **Table 2B**). Mice vaccinated with a high or a low dose of WT HA/NA protein without adjuvant lost ~10% of their original body weight, whereas mice vaccinated with a high or low dose of WT HA/NA plus adjuvant or high and low doses of COBRA HA/NA with or without adjuvant lost ~5% of their original body weight. All these vaccinated mice survived challenge (**Table 2B**). However, mice vaccinated with high-dose COBRA plus adjuvant were significantly protected against BR/18 challenge (**Supplementary Table 4A**). Mock-vaccinated mice and mice vaccinated with a low dose of unadjuvanted WT HA/NA proteins had, on average, viral lung titers >1×10^5^ PFU/g after H1N1 challenge (**Figure 8D**). There were no detectable viral lung titers in mice vaccinated with any of the other vaccine formulations, except one mouse in the low-dose unadjuvanted COBRA HA/NA.

Similar to the BR/18 H1N1 viral challenge, mock mice challenged with SW/13 H3N2 influenza virus lost ~25% of their original body weight by day 5 post-challenge (**Figure 7B**; **Supplementary Table 4B**), but 100% of mock mice did not survive challenge (**Figure 7E**) with average viral lung titers of ~1×10^5^ PFU/g (**Figure 8E**). Mice vaccinated with a high dose of either COBRA HA/NA with or without adjuvant and a high dose of WT HA/NA plus adjuvant lost less than 7% of their original body weight, with COBRA HA/NA plus adjuvant vaccinated mice losing, on average, less than 2% of their original body weight (**Figure 7B**). Mice vaccinated with other vaccine formulations had greater than 10% weight loss but only mice vaccinated with low-dose unadjuvanted COBRA HA/NA or low-dose unadjuvanted WT HA/NA proteins had less than 100% survival (**Figure 7E**; **Table 2B**). Only mice in the low-dose groups had detectable viral lung titers (**Figure 8E**).

Pre-immune mock-vaccinated mice that were challenged with the WA/19 IBV lost ~10% of their original weight and recovered with all mice surviving infection (**Figures 7C, F**). Mice vaccinated with any of the other vaccine formulations lost, on average, 5%–7% of their original weight at any timepoint post-challenge with all mice surviving challenge expect one mouse that appeared to be unrelated to the viral infection (**Figure 7F**; **Table 2B**). Only mock-vaccinated and low-dose unadjuvanted WT HA/NA had any detectable viral titers (**Figure 8F**).

## 4 Discussion

The ongoing antigenic evolution of influenza viruses, together with their capacity for reassortment, underscores the need for next-generation influenza vaccines capable of eliciting broader and more durable protective immune responses (29). Indeed, combined COBRA HA and NA recombinant proteins designed to represent circulating seasonal subtypes induce immune responses with enhanced breadth and protective capacity in animal models (11, 13). To further improve vaccine efficacy, multiple strategies have been investigated, including the use of adjuvants, optimization of prime-boost regimens, and alternative routes of administration, with the goal of enhancing the magnitude, quality, and durability of the immune response.

This study assessed the impact of c-di-AMP adjuvant on the immunogenicity and protective capacity of an intranasal pentavalent COBRA vaccine comprising HA (H1, H3, and IBV) and NA (N1 and N2) antigens across various dosing regimens in naïve and pre-immune models. Our findings demonstrate that the adjuvant shapes the quality of the immune response elicited by the pentavalent vaccine. While unadjuvanted formulations elicited high antibody titers, the inclusion of c-di-AMP shifted the response toward a more balanced Th1/Th2/Th17 profile (30). Animal models vaccinated with multivalent COBRA formulations, particularly when combined with adjuvants, elicit antibody responses and confer protection against viral challenge (12, 26, 31, 32). This study extends prior works by evaluating a novel multivalent COBRA formulation—H1 (Y2), H3 (NG7), IBV (BC17), N1 (N1I), and N2 (N2B)—with WT HA/NA comparators to assess the impact of vaccine dose and adjuvant on humoral and cellular immunity.

Intranasal vaccination with the pentavalent COBRA rHA/rNA formulation enhanced HA- and NA-specific systemic IgG in mice with pre-existing anti-influenza antibodies, suggesting improved antibody breadth and affinity. This finding aligns with prior studies demonstrating that COBRA-based vaccines induce broader and stronger antibody responses than strain-matched WT HA and NA antigens (22, 33). Mice vaccinated with 1 µg of COBRA antigens adjuvanted with c-di-AMP maintained higher antibody levels compared to unadjuvanted vaccinated mice showing a consistent two- to fourfold increase in IgG titers against all HA and NA antigens in the vaccine. Furthermore, c-di-AMP enabled the low-dose (0.1 µg) COBRA formulation to elicit significant IgG binding titers, confirming the antigen-sparing potential of this adjuvant against drifted strains (34, 35).

The rHA/rNA multivalent vaccines administered with c-di-AMP elicited robust IgG1 responses following vaccination in pre-immune mice (**Supplementary Figure 1**). Although the antibody subclass profile suggested an IgG1-skewed humoral immune response (IgG1 > IgG2a), mice vaccinated with an adjuvanted vaccine shifted the response toward a more balanced Th1/Th2 profile. This finding is consistent with the ability of c-di-AMP to enhance dendritic cell activation and promote mixed Th1/Th2/Th17 immunity (35, 36). The IgG1:IgG2a ratio is a critical indicator of the T-helper cell polarization (Th2 vs. Th1) induced by the immunization (37). This shift toward a more balanced Th1 (cellular/pro-inflammatory) and Th2 (humoral/antibody) subclass profile is clinically relevant; the increased IFN-γ production observed in our ELISpot analysis suggests that adjuvanted vaccination provides enhanced effector functions, likely contributing to the improved viral clearance we observed in challenged cohorts (38, 39).

Since immune history shapes antibody cross-reactivity to influenza A/H1N1, A/H3N2, and B viruses (40, 41), the study was designed with different two cohorts of naïve and previously exposed mice to historical H1N1, H3N2, and IBV to evaluate HAI titers. Naïve mice typically have comparatively lower baseline humoral responses compared to mice pre-exposed to historical influenza strains (42). Here, naïve mice immunized with the high-dose c-di-AMP–adjuvanted formulation developed protective HAI titers against most H1N1 strains and a subset of H3N2 strains, but not against IBV. This is consistent with previous work from our group showing that COBRA H1N1 antigens (e.g., Y2) induce broader HAI responses against H1 strains, while breadth against H3 and influenza B is more dependent on immune background (7–9). Clinically, pre-existing immunity influences the magnitude of serum antibody responses to influenza vaccination (43, 44). Indeed, pre-immunized mice had elevated HAI titers against all tested strains following 1 µg of rHA/rNA vaccination (**Figures 4D–F**), independent of adjuvant use. This is consistent with previous studies in pre-immune models (12, 14) and likely reflects the reactivation of HA-specific memory B cells, leading to robust B-cell expansion and broader HAI responses compared to naïve animals (45). The superior HAI responses observed in adjuvanted groups highlight the critical role adjuvant plays in boosting the potency of the multivalent COBRA platform, particularly in naïve hosts who lack pre-existing memory B-cell populations.

Because influenza virus vaccination is strongly influenced by pre-existing immunological memory (46), we assessed antibody-secreting cells’ production in mice previously infected with historical viruses. Mice vaccinated with 1 μg of rHA/rNA induced robust systemic ASC responses against HA and NA antigens. For H1N1, c-di-AMP likely increased the recruitment of ASCs (**Figure 5A**). This is consistent with the antibody data, where 1 µg of adjuvanted rHA/rNA induced higher HAI and IgG titers than formulations without adjuvant. However, in our study, all other antigens (H3, IBV, N1, and N2) did not require adjuvant to induce high frequencies of antigen-specific ASCs after multivalent vaccination. Higher antibody titers reflect expansion and diversification of the B-cell repertoire, correlating with increased antibody production and improved protection (47). Accordingly, ASCs expanding following vaccination is probably due to the memory B-cell activation after COBRA vaccination, regardless of adjuvant use. These findings highlight a direct link between B-cell dynamics and humoral responses following intranasal COBRA vaccination.

While COBRA HA/NA vaccination elicits broadly protective antibody responses, adjuvants are increasingly used to enhance vaccine-induced T-cell immunity (27, 48). It is well established that STING agonist-based adjuvants induce a balanced but strongly Th1-skewed cellular immune response (49, 50), whereas mucosal c-di-AMP promotes cellular immunity with a balanced Th1/Th2/Th17 profile (21, 51). In fact, our ELISpot analysis showed that prime-boost intranasal immunization with c-di-AMP-adjuvanted pentavalent vaccine induced high frequencies of antigen-specific IFN-γ- and IL-17-secreting splenic T cells, whereas unadjuvanted vaccination elicited minimal cellular responses.

Other studies have also shown that c-di-AMP induces robust antigen-specific IFN-γ- and IL-17-secreting splenocytes (27, 52). Interestingly, for H1 HA and IBV HA peptide pools, IL-17-secreting T-cell frequencies were lower in COBRA + c-di-AMP compared to WT + c-di-AMP groups (**Figures 1F, H**). This was likely driven by the differences in T-cell epitope composition between the COBRA consensus antigens and the WT peptide pools used for splenocytes stimulation. Possibly, COBRA-primed T cells recognize these peptides with reduced functional avidity, such that T-cell receptor (TCR) signaling becomes less favorable for IL-17 production while remaining sufficient to support IFN-γ responses (53). Mechanistically, CDNs promote dendritic cell maturation and activation, enhancing T cell-stimulatory capacity (54) through innate immune pathways, which induces IFN-γ production (55). This may underlie the enhanced T-cell responses and associated disease protection observed here.

Taken together, pre-immune animals vaccinated with c-di-AMP had higher antigen-specific IgG titers (**Figure 3**) and increased numbers of antigen-specific IFN-γ-secreting cells (**Figures 1A–E**; **Supplementary Figure 2A**) compared to unadjuvanted formulations. However, HAI titers were comparable between adjuvanted and unadjuvanted 1-µg COBRA formulations in this cohort (**Figures 4D–F**). Meanwhile, protective HAI titers against H1N1 influenza viruses were achieved with low-dose adjuvanted COBRA vaccination. A reduced dose-sparing effect observed for H3N2 and IBVs might be potentially due to antigen competition within the pentavalent mixture of the vaccine, as reported in other studies (34). The fact that adjuvant inclusion rescued these responses suggests that c-di-AMP helps overcome such competition by enhancing the activation and recruitment of APCs and improving antigen presentation efficiency (56), thereby ensuring a more balanced immune response across all five vaccine components.

Challenge of both immunologically naïve and pre-immune DBA/2J mice showed that stronger immune responses and complete protection required either a higher vaccine dose or adjuvant use. In the naïve cohort, adjuvanted COBRA vaccines induced broad HAI titers against H1N1 even at lower doses, correlating with protection from severe disease (**Figures 6A–C**) and lower viral load in lungs (**Figures 8A–C**). Pre-existing immunity also influenced HAI antibody titers for H1N1, H3N2, and IBV (**Figure 4**), disease protection (**Figures 6**, **7**; **Table 2**), and viral replication in the lungs (**Figures 8A–C**). This may be driven by cross-reactive antibody and T-cell responses generated from prior exposure to historical influenza strains (3, 57). The comprehensive protection characterized by survival and significantly reduced pulmonary viral replications is not merely an outcome of higher antibody titers, but a functional consequence of the adjuvant-induced immune profile. We propose that the combination of Th1/Th2-balanced responses and high HAI titers induced by the adjuvanted COBRA vaccine is sufficient to protect naïve mice from influenza disease and mortality.

Notably, pre-immune mice vaccinated with 1 µg of COBRA HA/NA proteins without adjuvant had high HAI titers and survived infection, consistent with increased ASC responses, suggesting that the COBRA antigens alone are highly effective at inducing antibody responses. On the other hand, the adjuvanted pentavalent COBRA formulation induced higher frequency of IFN-γ production by Th1-type cells compared to the unadjuvanted vaccine (**Figure 1**). These cells are increasingly recognized as key mediators of rapid viral control and heterologous protection, reducing influenza severity (58). Consistently, the enhanced humoral and cellular responses in the high-dose adjuvanted groups translated into functional protection following H1N1, H3N2, and IBV challenge (**Figures 6**, **7**; **Table 2**). This underscores that for multivalent COBRA vaccines, the adjuvant is not just a dose-sparing tool, but a critical determinant of protective breadth against antigenically distinct influenza strains.

This study provides a framework for optimizing multivalent COBRA intranasal vaccines in terms of antigen dose, pre-existing immune history, and adjuvant use, and shows their effects on systemic humoral and cellular immune responses as well as functional protection following lethal challenge. While we focused on systemic correlates of immunity, local mucosal immune responses in the respiratory tract were not directly measured. Intranasal delivery of vaccine formulated with c-di-AMP likely engages the mucosal compartment through induction of IgA responses in the respiratory tract (35, 50); therefore, the absence of such readouts in the present study should be considered a limitation. Importantly, the aim of the current study was not to evaluate mechanisms of mucosal responses, but rather to assess the efficacy of intranasal delivery of multivalent COBRA recombinant HA and NA proteins formulated with an adjuvant in eliciting broadly cross-protective antibody, B-cell, and T-cell responses. Future work will directly assess mucosal correlates, including nasal wash IgA and tissue-resident memory B- and T-cell populations in the respiratory tract, to define the local mechanisms underlying the cross-protective immunity observed here.

Collectively, these findings show that this needle-free, intranasally delivered pentavalent COBRA HA/NA vaccine platform formulated with c-di-AMP broadens humoral and cellular immunity against seasonal influenza A and B viruses in a dose-dependent manner. The immune response enhances cross-subtype protection, limits viral replication, and reduces disease severity in both naïve and pre-immune mice. These vaccine strategies may be well-suited for protection against diverse influenza virus subtypes, with the potential to reduce strain mismatch across distinct immune backgrounds. Further optimization of this strategy may enable cross-protective immunity against current and emerging influenza viruses.

## Data Availability

The original contributions presented in the study are included in the article/**Supplementary Material**. Further inquiries can be directed to the corresponding author.
