# Supplementary material for: Intranasal delivery of a COBRA-based protein vaccine adjuvanted with c-di-AMP enhances breadth of protective immune responses against seasonal influenza viruses
Source: Front Immunol. 2026 Aug 21;17:1876199. doi: 10.3389/fimmu.2026.1876199 (PMC13541677; doi:10.3389/fimmu.2026.1876199)
Supplement: Supplementary file 1 [file DataSheet1.docx]

Supplementary Material

**Supplementary Figures**

**A**

**B**

**Supplementary Figure 1. Serum IgG1:IgG2a ratios in pre-immune mice following intranasal vaccination.** Mice were vaccinated with different concentrations of COBRA or wild type HA and NA vaccines with c-di-AMP or no adjuvant. Sera were collected 14 days after the second vaccination from pre-immune mice. The proportion of serum IgG1 and IgG2a from vaccinated pre-immune mice is depicted. Ratios IgG1:IgG2a are provided for responses against COBRA and wild type influenza antigens. The values represent the ratio of IgG1 Area Under the Curve (AUC) to IgG2a AUC. Data is presented for seven experimental groups (G1–G7) as defined in the key. G1: COBRA (1ug) + c-di-AMP; G2: COBRA (0.1ug) + c-di-AMP; G3: COBRA (1ug); G4: WT (1ug) + c-di-AMP; G5: WT (0.1ug) + c-di-AMP; G6: WT (1ug); G7: PBS + c-di-AMP.

**Supplementary Figure 2. Quantification of IFN-γ and IL-17 secreting cells per vaccine group following stimulation with HA/NA head and stem peptide pools.** Splenocytes from immunized mice were stimulated *ex vivo* with peptide pools corresponding to the "Head" or "Stem" regions of influenza hemagglutinin (HA) (H1N1, H3N2, IBV) and neuraminidase (NA) (H1N1, H3N2). Data represent the frequency of IFN-γ and IL-17 secreting cells per million splenocytes for three representative groups: COBRA (1μg) + c-di-AMP, COBRA (1μg) and Wild Type (1μg) + c-di-AMP.

**Supplementary Tables**

**Supplementary Table 1. Statistical differences in HAI Titers after vaccination in the naïve cohort.** Statistical differences of HAI titers between vaccine groups in naïve animals for H1N1 **(Fig. 3A)**, H3N2 **(Fig. 3B)** viruses were analyzed using Kruskal–Wallis one-way ANOVA with Dunn's multiple comparisons with GraphPad Prism 9 software (GraphPad, San Diego, CA, USA). A p value of less than 0.05 was defined as statistically significant (*, p < 0.05; **, p < 0.01; ***, p < 0.001; ****, p < 0.0001). No statistically significant differences were observed in HAI titers among the influenza B (IBV) groups. No applicable (N/A) means the virus strain did not elicit detectable antibodies.

**A**

| **H1N1 Comparison** | **BR/18** | **GD/19** | **VIC/19** |
| --- | --- | --- | --- |
| 1ug COBRA + c-di-AMP     vs. 0.1ug COBRA + c-di-AMP | ns | ns | *** |
| 1ug COBRA + c-di-AMP     vs. 1ug COBRA | **** | *** | *** |
| 1ug COBRA + c-di-AMP     vs. 0.1ug COBRA | **** | *** | *** |
| 1ug COBRA + c-di-AMP     vs. 1ug WT + c-di-AMP | * | ns | ns |
| 1ug COBRA + c-di-AMP     vs. 0.1ug WT + c-di-AMP | **** | *** | ns |
| 1ug COBRA + c-di-AMP     vs. 1ug WT | **** | *** | ns |
| 1ug COBRA + c-di-AMP     vs. 0.1ug WT | **** | *** | *** |
| 1ug COBRA + c-di-AMP     vs. PBS + c-di-AMP | **** | *** | *** |
| 0.1ug COBRA + c-di-AMP vs. 1ug COBRA | ns | ns | ns |
| 0.1ug COBRA + c-di-AMP vs. 0.1ug COBRA | ns | ns | ns |
| 0.1ug COBRA + c-di-AMP vs. 1ug WT + c-di-AMP | ns | ns | ns |
| 0.1ug COBRA + c-di-AMP vs. 0.1ug WT + c-di-AMP | ns | ns | ns |
| 0.1ug COBRA + c-di-AMP vs. 1ug WT | ns | ns | ns |
| 0.1ug COBRA + c-di-AMP vs. 0.1ug WT | ns | ns | ns |
| 0.1ug COBRA + c-di-AMP vs. PBS + c-di-AMP | ns | ns | ns |
| 1ug COBRA vs. 0.1ug COBRA | ns | ns | ns |
| 1ug COBRA vs. 1ug WT + c-di-AMP | ns | ns | ns |
| 1ug COBRA vs. 0.1ug WT + c-di-AMP | ns | ns | ns |
| 1ug COBRA vs. 1ug WT | ns | ns | ns |
| 1ug COBRA vs. 0.1ug WT | ns | ns | ns |
| 1ug COBRA vs. PBS + c-di-AMP | ns | ns | ns |
| 0.1ug COBRA vs. 1ug WT + c-di-AMP | ns | ns | ns |
| 0.1ug COBRA vs. 0.1ug WT + c-di-AMP | ns | ns | ns |
| 0.1ug COBRA vs. 1ug WT | ns | ns | ns |
| 0.1ug COBRA vs. 0.1ug WT | ns | ns | ns |
| 0.1ug COBRA vs. PBS + c-di-AMP | ns | ns | ns |
| 1ug WT + c-di-AMP vs. 0.1ug WT + c-di-AMP | ns | ns | ns |
| 1ug WT + c-di-AMP vs. 1ug WT | ns | ns | ns |
| 1ug WT + c-di-AMP vs. 0.1ug WT | ns | ns | ns |
| 1ug WT + c-di-AMP vs. PBS + c-di-AMP | ns | ns | ns |
| 0.1ug WT + c-di-AMP vs. 1ug WT | ns | ns | ns |
| 0.1ug WT + c-di-AMP vs. 0.1ug WT | ns | ns | ns |
| 0.1ug WT + c-di-AMP vs. PBS + c-di-AMP | ns | ns | ns |
| 1ug WT vs. 0.1ug WT | ns | ns | ns |
| 1ug WT vs. PBS + c-di-AMP | ns | ns | ns |
| 0.1ug WT vs. PBS + c-di-AMP | ns | ns | ns |

**B**

| **H3N2 Comparison** | **SW/13** | **SA/19** | **HK/19** | **TAS/20** |
| --- | --- | --- | --- | --- |
| 1ug COBRA + c-di-AMP     vs. 0.1ug COBRA + c-di-AMP | N/A | **** | ns | N/A |
| 1ug COBRA + c-di-AMP     vs. 1ug COBRA | N/A | **** | ns | N/A |
| 1ug COBRA + c-di-AMP     vs. 0.1ug COBRA | N/A | **** | ** | N/A |
| 1ug COBRA + c-di-AMP     vs. 1ug WT + c-di-AMP | N/A | ns | ns | N/A |
| 1ug COBRA + c-di-AMP     vs. 0.1ug WT + c-di-AMP | N/A | **** | ** | N/A |
| 1ug COBRA + c-di-AMP     vs. 1ug WT | N/A | **** | ns | N/A |
| 1ug COBRA + c-di-AMP     vs. 0.1ug WT | N/A | **** | * | N/A |
| 1ug COBRA + c-di-AMP     vs. PBS + c-di-AMP | N/A | **** | ** | N/A |
| 0.1ug COBRA + c-di-AMP vs. 1ug COBRA | N/A | ns | ns | N/A |
| 0.1ug COBRA + c-di-AMP vs. 0.1ug COBRA | N/A | ns | ns | N/A |
| 0.1ug COBRA + c-di-AMP vs. 1ug WT + c-di-AMP | N/A | ns | ns | N/A |
| 0.1ug COBRA + c-di-AMP vs. 0.1ug WT + c-di-AMP | N/A | ns | ns | N/A |
| 0.1ug COBRA + c-di-AMP vs. 1ug WT | N/A | ns | ns | N/A |
| 0.1ug COBRA + c-di-AMP vs. 0.1ug WT | N/A | ns | ns | N/A |
| 0.1ug COBRA + c-di-AMP vs. PBS + c-di-AMP | N/A | ns | ns | N/A |
| 1ug COBRA vs. 0.1ug COBRA | N/A | ns | ** | N/A |
| 1ug COBRA vs. 1ug WT + c-di-AMP | N/A | ns | ns | N/A |
| 1ug COBRA vs. 0.1ug WT + c-di-AMP | N/A | ns | *** | N/A |
| 1ug COBRA vs. 1ug WT | N/A | ns | ns | N/A |
| 1ug COBRA vs. 0.1ug WT | N/A | ns | ** | N/A |
| 1ug COBRA vs. PBS + c-di-AMP | N/A | ns | *** | N/A |
| 0.1ug COBRA vs. 1ug WT + c-di-AMP | N/A | ns | ns | N/A |
| 0.1ug COBRA vs. 0.1ug WT + c-di-AMP | N/A | ns | ns | N/A |
| 0.1ug COBRA vs. 1ug WT | N/A | ns | * | N/A |
| 0.1ug COBRA vs. 0.1ug WT | N/A | ns | ns | N/A |
| 0.1ug COBRA vs. PBS + c-di-AMP | N/A | ns | ns | N/A |
| 1ug WT + c-di-AMP vs. 0.1ug WT + c-di-AMP | N/A | ns | ns | N/A |
| 1ug WT + c-di-AMP vs. 1ug WT | N/A | ns | ns | N/A |
| 1ug WT + c-di-AMP vs. 0.1ug WT | N/A | ns | ns | N/A |
| 1ug WT + c-di-AMP vs. PBS + c-di-AMP | N/A | ns | ns | N/A |
| 0.1ug WT + c-di-AMP vs. 1ug WT | N/A | ns | ** | N/A |
| 0.1ug WT + c-di-AMP vs. 0.1ug WT | N/A | ns | ns | N/A |
| 0.1ug WT + c-di-AMP vs. PBS + c-di-AMP | N/A | ns | ns | N/A |
| 1ug WT vs. 0.1ug WT | N/A | ns | ns | N/A |
| 1ug WT vs. PBS + c-di-AMP | N/A | ns | ** | N/A |
| 0.1ug WT vs. PBS + c-di-AMP | N/A | ns | ns | N/A |

**Supplementary Table 2. Statistical differences in HAI Titers after vaccination in the pre-immune cohort.** Statistical differences of HAI titers between vaccine groups in pre-immune animals for H1N1 **(Fig. 3A)**, H3N2 **(Fig. 3B)** and influenza B **(Fig. 3C)** viruses were analyzed using Kruskal–Wallis one-way ANOVA with Dunn's multiple comparisons with GraphPad Prism 9 software (GraphPad, San Diego, CA, USA). A p value of less than 0.05 was defined as statistically significant (*, p < 0.05; **, p < 0.01; ***, p < 0.001; ****, p < 0.0001).

**A**

| **H1N1 Comparison** | **BR/18** | **GD/19** | **VIC/19** |
| --- | --- | --- | --- |
| 1ug COBRA + c-di-AMP     vs. 0.1ug COBRA + c-di-AMP | ns | ns | ns |
| 1ug COBRA + c-di-AMP     vs. 1ug COBRA | ns | ns | ns |
| 1ug COBRA + c-di-AMP     vs. 0.1ug COBRA | *** | ns | ** |
| 1ug COBRA + c-di-AMP     vs. 1ug WT + c-di-AMP | ns | ns | ns |
| 1ug COBRA + c-di-AMP     vs. 0.1ug WT + c-di-AMP | ** | ns | *** |
| 1ug COBRA + c-di-AMP     vs. 1ug WT | ns | ns | ns |
| 1ug COBRA + c-di-AMP     vs. 0.1ug WT | **** | ** | * |
| 1ug COBRA + c-di-AMP     vs. PBS + c-di-AMP | **** | * | *** |
| 0.1ug COBRA + c-di-AMP vs. 1ug COBRA | ns | ns | ns |
| 0.1ug COBRA + c-di-AMP vs. 0.1ug COBRA | ns | ns | ns |
| 0.1ug COBRA + c-di-AMP vs. 1ug WT + c-di-AMP | ns | ns | ns |
| 0.1ug COBRA + c-di-AMP vs. 0.1ug WT + c-di-AMP | ns | ns | ns |
| 0.1ug COBRA + c-di-AMP vs. 1ug WT | ns | ns | ns |
| 0.1ug COBRA + c-di-AMP vs. 0.1ug WT | ns | ns | ns |
| 0.1ug COBRA + c-di-AMP vs. PBS + c-di-AMP | ns | ns | ns |
| 1ug COBRA vs. 0.1ug COBRA | * | ns | ns |
| 1ug COBRA vs. 1ug WT + c-di-AMP | ns | ns | ns |
| 1ug COBRA vs. 0.1ug WT + c-di-AMP | ns | ns | * |
| 1ug COBRA vs. 1ug WT | ns | ns | ns |
| 1ug COBRA vs. 0.1ug WT | ** | ns | ns |
| 1ug COBRA vs. PBS + c-di-AMP | ** | ns | ** |
| 0.1ug COBRA vs. 1ug WT + c-di-AMP | ** | ns | ** |
| 0.1ug COBRA vs. 0.1ug WT + c-di-AMP | ns | ns | ns |
| 0.1ug COBRA vs. 1ug WT | ns | ns | * |
| 0.1ug COBRA vs. 0.1ug WT | ns | ns | ns |
| 0.1ug COBRA vs. PBS + c-di-AMP | ns | ns | ns |
| 1ug WT + c-di-AMP vs. 0.1ug WT + c-di-AMP | * | ns | *** |
| 1ug WT + c-di-AMP vs. 1ug WT | ns | ns | ns |
| 1ug WT + c-di-AMP vs. 0.1ug WT | *** | ns | * |
| 1ug WT + c-di-AMP vs. PBS + c-di-AMP | *** | ns | *** |
| 0.1ug WT + c-di-AMP vs. 1ug WT | ns | ns | ** |
| 0.1ug WT + c-di-AMP vs. 0.1ug WT | ns | ns | ns |
| 0.1ug WT + c-di-AMP vs. PBS + c-di-AMP | ns | ns | ns |
| 1ug WT vs. 0.1ug WT | * | ns | ns |
| 1ug WT vs. PBS + c-di-AMP | * | ns | ** |
| 0.1ug WT vs. PBS + c-di-AMP | ns | ns | ns |

**B**

| **H3N2 Comparison** | **SW/13** | **SA/19** | **HK/19** | **TAS/20** |
| --- | --- | --- | --- | --- |
| 1ug COBRA + c-di-AMP     vs. 0.1ug COBRA + c-di-AMP | * | ns | ns | ns |
| 1ug COBRA + c-di-AMP     vs. 1ug COBRA | ns | ns | ns | ns |
| 1ug COBRA + c-di-AMP     vs. 0.1ug COBRA | * | * | ns | **** |
| 1ug COBRA + c-di-AMP     vs. 1ug WT + c-di-AMP | ns | ns | ns | ns |
| 1ug COBRA + c-di-AMP     vs. 0.1ug WT + c-di-AMP | ns | * | ns | * |
| 1ug COBRA + c-di-AMP     vs. 1ug WT | ns | ns | ns | ns |
| 1ug COBRA + c-di-AMP     vs. 0.1ug WT | * | *** | ns | **** |
| 1ug COBRA + c-di-AMP     vs. PBS + c-di-AMP | * | *** | ns | * |
| 0.1ug COBRA + c-di-AMP vs. 1ug COBRA | ns | ns | ns | ns |
| 0.1ug COBRA + c-di-AMP vs. 0.1ug COBRA | ns | ns | ns | ns |
| 0.1ug COBRA + c-di-AMP vs. 1ug WT + c-di-AMP | ns | ns | ns | ns |
| 0.1ug COBRA + c-di-AMP vs. 0.1ug WT + c-di-AMP | ns | ns | ns | ns |
| 0.1ug COBRA + c-di-AMP vs. 1ug WT | ns | ns | ns | ns |
| 0.1ug COBRA + c-di-AMP vs. 0.1ug WT | ns | ns | ns | ns |
| 0.1ug COBRA + c-di-AMP vs. PBS + c-di-AMP | ns | ns | ns | ns |
| 1ug COBRA vs. 0.1ug COBRA | ns | ns | ns | *** |
| 1ug COBRA vs. 1ug WT + c-di-AMP | ns | ns | ns | ns |
| 1ug COBRA vs. 0.1ug WT + c-di-AMP | ns | * | ns | ns |
| 1ug COBRA vs. 1ug WT | ns | ns | ns | ns |
| 1ug COBRA vs. 0.1ug WT | ns | ** | ** | *** |
| 1ug COBRA vs. PBS + c-di-AMP | ns | ** | ** | ns |
| 0.1ug COBRA vs. 1ug WT + c-di-AMP | ns | * | ns | ** |
| 0.1ug COBRA vs. 0.1ug WT + c-di-AMP | ns | ns | ns | ns |
| 0.1ug COBRA vs. 1ug WT | ns | ns | ns | * |
| 0.1ug COBRA vs. 0.1ug WT | ns | ns | ns | ns |
| 0.1ug COBRA vs. PBS + c-di-AMP | ns | ns | ns | ns |
| 1ug WT + c-di-AMP vs. 0.1ug WT + c-di-AMP | ns | * | ns | ns |
| 1ug WT + c-di-AMP vs. 1ug WT | ns | ns | ns | ns |
| 1ug WT + c-di-AMP vs. 0.1ug WT | ns | ** | ns | ** |
| 1ug WT + c-di-AMP vs. PBS + c-di-AMP | ns | ** | ns | ns |
| 0.1ug WT + c-di-AMP vs. 1ug WT | ns | ns | ns | ns |
| 0.1ug WT + c-di-AMP vs. 0.1ug WT | ns | ns | ns | ns |
| 0.1ug WT + c-di-AMP vs. PBS + c-di-AMP | ns | ns | ns | ns |
| 1ug WT vs. 0.1ug WT | ns | * | ns | * |
| 1ug WT vs. PBS + c-di-AMP | ns | * | ns | ns |
| 0.1ug WT vs. PBS + c-di-AMP | ns | ns | ns | ns |

**C**

| **IBV Comparison** | **B/BR/08** | **B/CO/17** | **B/WA/19** |
| --- | --- | --- | --- |
| 1ug COBRA + c-di-AMP     vs. 0.1ug COBRA + c-di-AMP | ns | ns | ns |
| 1ug COBRA + c-di-AMP     vs. 1ug COBRA | ns | ns | ns |
| 1ug COBRA + c-di-AMP     vs. 0.1ug COBRA | ns | ns | ** |
| 1ug COBRA + c-di-AMP     vs. 1ug WT + c-di-AMP | * | ns | * |
| 1ug COBRA + c-di-AMP     vs. 0.1ug WT + c-di-AMP | ** | ** | *** |
| 1ug COBRA + c-di-AMP     vs. 1ug WT | ns | ns | ** |
| 1ug COBRA + c-di-AMP     vs. 0.1ug WT | **** | **** | **** |
| 1ug COBRA + c-di-AMP     vs. PBS + c-di-AMP | ns | ns | ns |
| 0.1ug COBRA + c-di-AMP vs. 1ug COBRA | ns | ns | ns |
| 0.1ug COBRA + c-di-AMP vs. 0.1ug COBRA | ns | ns | ns |
| 0.1ug COBRA + c-di-AMP vs. 1ug WT + c-di-AMP | ns | ns | ns |
| 0.1ug COBRA + c-di-AMP vs. 0.1ug WT + c-di-AMP | ns | ns | ns |
| 0.1ug COBRA + c-di-AMP vs. 1ug WT | ns | ns | ns |
| 0.1ug COBRA + c-di-AMP vs. 0.1ug WT | * | ns | ns |
| 0.1ug COBRA + c-di-AMP vs. PBS + c-di-AMP | ns | ns | ns |
| 1ug COBRA vs. 0.1ug COBRA | ns | ns | ns |
| 1ug COBRA vs. 1ug WT + c-di-AMP | ns | ns | ns |
| 1ug COBRA vs. 0.1ug WT + c-di-AMP | * | ** | * |
| 1ug COBRA vs. 1ug WT | ns | ns | ns |
| 1ug COBRA vs. 0.1ug WT | **** | *** | *** |
| 1ug COBRA vs. PBS + c-di-AMP | ns | ns | ns |
| 0.1ug COBRA vs. 1ug WT + c-di-AMP | ns | ns | ns |
| 0.1ug COBRA vs. 0.1ug WT + c-di-AMP | ns | ns | ns |
| 0.1ug COBRA vs. 1ug WT | ns | ns | ns |
| 0.1ug COBRA vs. 0.1ug WT | ns | ns | ns |
| 0.1ug COBRA vs. PBS + c-di-AMP | ns | ns | ns |
| 1ug WT + c-di-AMP vs. 0.1ug WT + c-di-AMP | ns | ns | ns |
| 1ug WT + c-di-AMP vs. 1ug WT | ns | ns | ns |
| 1ug WT + c-di-AMP vs. 0.1ug WT | ns | ns | ns |
| 1ug WT + c-di-AMP vs. PBS + c-di-AMP | ns | ns | ns |
| 0.1ug WT + c-di-AMP vs. 1ug WT | ns | ns | ns |
| 0.1ug WT + c-di-AMP vs. 0.1ug WT | ns | ns | ns |
| 0.1ug WT + c-di-AMP vs. PBS + c-di-AMP | ns | ns | ns |
| 1ug WT vs. 0.1ug WT | ns | ns | ns |
| 1ug WT vs. PBS + c-di-AMP | ns | ns | ns |
| 0.1ug WT vs. PBS + c-di-AMP | ns | ns | ns |

**Supplementary Table 3. Statistical differences in body weight loss after virus challenge in naive mice after (A) A/BR/18 (B) A/SW/13 or WA/19 challenge.** Statistical differences in weight loss between vaccine groups from day 1 to day 12 after challenge (Figure 6) were analyzed using Kruskal-Wallis ANOVA with Dunn’s multiple comparison with GraphPad Prism 9 software (GraphPad, San Diego, CA, USA). A p value of less than 0.05 was defined as statistically significant (*, p < 0.05; **, p < 0.01; ***, p < 0.001; ****, p < 0.0001). No statistically significant differences in body weight were observed among the influenza B (IBV) challenge groups.

**A**

| **H1N1 Comparison** | **Day 1** | **Day 2** | **Day 3** | **Day 4** | **Day 5** | **Day 6** | **Day 7** | **Day 8** | **Day 9** | **Day 10** | **Day 11** | **Day 12** |
| --- | --- | --- | --- | --- | --- | --- | --- | --- | --- | --- | --- | --- |
| 1ug COBRA + c-di-AMP vs. 1ug COBRA | ns | ns | * | ns | ns | ns | ns | ns | ns | ns | ns | ns |
| 1ug COBRA + c-di-AMP vs. 0.1ug COBRA | ns | ns | * | * | ns | ns | ns | ns | ns | ns | ns | ns |
| 1ug COBRA + c-di-AMP vs. 0.1ug WT + c-di-AMP | ns | ns | **** | ns | ns | ns | ns | ns | ns | ns | ns | ns |
| 1ug COBRA + c-di-AMP vs. 0.1ug WT | ns | ns | ** | * | ns | ns | ns | ns | ns | ns | ns | ns |
| 0.1ug COBRA + c-di-AMP vs. 1ug WT | ** | ns | ns | ns | ns | ns | ns | ns | ns | ns | ns | ns |
| 0.1ug COBRA + c-di-AMP vs. PBS + c-di-AMP | ns | * | ns | ns | ns | ns | ns | ns | ns | ns | ns | ns |
| 1ug WT + c-di-AMP vs. 0.1ug WT + c-di-AMP | ns | ns | ** | ns | ns | ns | ns | ns | ns | ns | ns | ns |
| 1ug WT + c-di-AMP vs. 1ug WT | * | ns | ns | ns | ns | ns | ns | ns | ns | ns | ns | ns |
| 1ug WT + c-di-AMP vs. PBS + c-di-AMP | ns | * | ns | ns | ns | ns | ns | ns | ns | ns | ns | ns |
| 0.1ug WT + c-di-AMP vs. 1ug WT | ns | ns | ** | ns | ns | ns | ns | ns | ns | ns | ns | ns |

**B**

| **H3N2 Comparison** | **Day 1** | **Day 2** | **Day 3** | **Day 4** | **Day 5** | **Day 6** | **Day 7** | **Day 8** | **Day 9** | **Day 10** | **Day 11** | **Day 12** |
| --- | --- | --- | --- | --- | --- | --- | --- | --- | --- | --- | --- | --- |
| 1ug COBRA + c-di-AMP vs. 0.1ug COBRA + c-di-AMP | ns | ns | ** | ns | ns | ns | ns | ns | ns | ns | ns | ns |
| 1ug COBRA + c-di-AMP vs. 0.1ug COBRA | ** | *** | ** | * | ns | ns | ns | ns | ns | ns | ns | ns |
| 1ug COBRA + c-di-AMP vs. 0.1ug WT + c-di-AMP | ns | ns | ** | ns | ns | ns | ns | ns | ns | ns | ns | ns |
| 0.1ug COBRA + c-di-AMP vs. 1ug WT + c-di-AMP | ns | ns | * | ns | ns | ns | ns | ns | ns | ns | ns | ns |
| 0.1ug COBRA vs. 1ug WT + c-di-AMP | ns | ns | * | ns | ns | ns | ns | ns | ns | ns | ns | ns |
| 0.1ug COBRA vs. PBS + c-di-AMP | * | ns | ns | ns | ns | ns | ns | ns | ns | ns | ns | ns |
| 1ug WT + c-di-AMP vs. 0.1ug WT + c-di-AMP | ns | ns | * | ns | * | ns | ns | ns | ns | ns | ns | ns |
| 1ug WT + c-di-AMP vs. 0.1ug WT | ns | ns | ns | ns | ns | ns | * | ns | ns | ns | ns | ns |

**Supplementary Table 4: Statistical differences in body weight loss after virus challenge in pre-immune mice after (A) A/BR/18 (B) A/SW/13 or WA/19 challenge.** Statistical differences in weight loss between vaccine groups from day 1 to day 12 after challenge (Figure 7) were analyzed using Kruskal-Wallis ANOVA with Dunn’s multiple comparison with GraphPad Prism 9 software (GraphPad, San Diego, CA, USA). A p value of less than 0.05 was defined as statistically significant (*, p < 0.05; **, p < 0.01; ***, p < 0.001; ****, p < 0.0001). No statistically significant differences in body weight were observed among the influenza B (IBV) challenge groups.

**A**

| **H1N1 Comparison** | **Day 1** | **Day 2** | **Day 3** | **Day 4** | **Day 5** | **Day 6** | **Day 7** | **Day 8** | **Day 9** | **Day 10** | **Day 11** | **Day 12** |
| --- | --- | --- | --- | --- | --- | --- | --- | --- | --- | --- | --- | --- |
| 1ug COBRA + c-di-AMP     vs. PBS + c-di-AMP | ns | ns | * | ** | * | ns | ns | ns | ns | ns | ns | ns |
| 1ug COBRA vs. 0.1ug WT | ns | * | ns | ns | ns | ns | ns | ns | ns | ns | ns | ns |

**B**

| **H3N2 Comparison** | **Day 1** | **Day 2** | **Day 3** | **Day 4** | **Day 5** | **Day 6** | **Day 7** | **Day 8** | **Day 9** | **Day 10** | **Day 11** | **Day 12** |
| --- | --- | --- | --- | --- | --- | --- | --- | --- | --- | --- | --- | --- |
| 1ug COBRA + c-di-AMP     vs. 0.1ug COBRA + c-di-AMP | ns | *** | ns | ns | ns | ns | ns | ns | ns | ns | * | ns |
| 1ug COBRA + c-di-AMP     vs. 0.1ug COBRA | ns | ns | ** | * | ns | * | ns | * | ns | ns | ns | ns |
| 1ug COBRA + c-di-AMP     vs. 0.1ug WT + c-di-AMP | ns | ** | * | ns | ns | ns | ns | ns | ns | ns | ns | ns |
| 1ug COBRA + c-di-AMP     vs. 0.1ug WT | ns | * | * | ns | ns | ns | ns | ns | ns | ns | ns | ns |
| 1ug COBRA + c-di-AMP     vs. PBS + c-di-AMP | ns | *** | **** | ** | ns | ns | ns | ns | ns | ns | ns | ns |
| 0.1ug COBRA + c-di-AMP vs. 1ug WT | ns | * | ns | ns | ns | ns | ns | ns | ns | ns | ns | ns |
| 0.1ug COBRA vs. 1ug WT | ns | ns | ns | ns | ns | * | ns | ns | ns | ns | ns | ns |
| 1ug WT + c-di-AMP vs. PBS + c-di-AMP | ns | ns | * | ns | ns | ns | ns | ns | ns | ns | ns | ns |
| 1ug WT vs. PBS + c-di-AMP | ns | * | * | ns | ns | ns | ns | ns | ns | ns | ns | ns |
